# Characterization of the landscape of the intratumoral microbiota reveals that *Streptococcus anginosus* increases the risk of gastric cancer initiation and progression

**DOI:** 10.1038/s41421-024-00746-0

**Published:** 2024-11-26

**Authors:** Li Yuan, Libin Pan, Yunzhe Wang, Jing Zhao, Luo Fang, Ying Zhou, Ruihong Xia, Yubo Ma, Zhengchen Jiang, Zhiyuan Xu, Can Hu, Yanan Wang, Shengjie Zhang, Bo Zhang, Haiying Ding, Mengxuan Chen, Haibo Cheng, Ajay Goel, Zhao Zhang, Xiangdong Cheng

**Affiliations:** 1 Department of Integrated Chinese and Western Medicine, Zhejiang Cancer Hospital, Hangzhou Institute of Medicine (HIM), Chinese Academy of Sciences, Hangzhou, Zhejiang China; 2 Zhejiang Provincial Research Center for Upper Gastrointestinal Tract Cancer, Zhejiang Cancer Hospital, Hangzhou, Zhejiang, China; 3 Zhejiang Key Lab of Prevention, Diagnosis and Therapy of Upper Gastrointestinal Cancer, Zhejiang Cancer Hospital, Hangzhou, Zhejiang, China; 4 Department of Pharmacy, Zhejiang Cancer Hospital, Hangzhou Institute of Medicine (HIM), Chinese Academy of Sciences, Hangzhou, Zhejiang China; 5 MOE Key Laboratory of Metabolism and Molecular Medicine, Department of Biochemistry and Molecular Biology, School of Basic Medical Sciences, Fudan University, Shanghai, China; 6 Department of Gastric Surgery, Zhejiang Cancer Hospital, Hangzhou Institute of Medicine (HIM), Chinese Academy of Sciences, Hangzhou, Zhejiang China; 7 Shanghai Analytical Applications Center, Shimadzu (China) Co., LTD, Shanghai, China; 8 The First School of Clinical Medicine, Nanjing University of Chinese Medicine, Nanjing, Jiangsu, China; 9 Department of Molecular Diagnostics and Experimental Therapeutics, Beckman Research Institute of City of Hope Comprehensive Cancer Center, Duarte, CA USA

**Keywords:** Gastric cancer, Tumour immunology, Bioinformatics

## Abstract

As a critical component of the tumour immune microenvironment (TIME), the resident microbiota promotes tumorigenesis across a variety of cancer types. Here, we integrated multiple types of omics data, including microbiome, transcriptome, and metabolome data, to investigate the functional role of intratumoral bacteria in gastric cancer (GC). The microbiome was used to categorize GC samples into six subtypes, and patients with a high abundance of *Streptococcus* or *Pseudomonas* had a markedly worse prognosis. Further assays revealed that *Streptococcus anginosus* (SA) promoted tumour cell proliferation and metastasis while suppressing the differentiation and infiltration of CD8^+^ T cells. However, antibiotic treatment significantly suppressed tumorigenesis in SA^+^ mice in vivo. We further demonstrated that the SA arginine pathway increased the abundance of ornithine, which may be a major contributor to reshaping of the TIME. Our findings demonstrated that SA, a novel risk factor, plays significant roles in the initiation and progression of GC, suggesting that SA might be a promising target for the diagnosis and treatment of GC.

## Introduction

Gastric cancer (GC) is one of the most common malignant tumours worldwide and is the fourth leading cause of cancer-related deaths. According to the 2000 data from the International Agency for Research on Cancer, there are 1.09 million newly diagnosed cases of GC worldwide annually, with 770,000 fatalities, posing a severe threat to human life and health^1^. The recognized epidemiological risk factors for GC mainly include *Helicobacter pylori* (HP) infection, age, high salt intake, and a diet low in fruits and vegetables^1^. It is well established that HP infection plays a role in the early steps of this process^2^. However, even though >50% of the global population is colonized with HP, only approximately 1%–3% of those individuals will ultimately develop GC^3^. In addition, eradicating HP to treat GC has yielded mixed results. Most studies suggest that eradicating HP successfully halts the progression of gastritis^4,5^. However, in some prospective trials, successful eradication of HP did not reduce the incidence of GC^6^. Thus, our understanding of the role of HP in GC might merely scratch the surface in terms of the role of the gastric microbiota in the development of tumours.

With continuous advancements in polymerase chain reaction (PCR) technology and metagenomics, an increasing body of research has shown that the stomach contains a robust microbiota^7^. Coker et al. demonstrated that, in comparison to the microbiota present in the precancerous stages, the GC microbiome is significantly enriched in *Parvimonas micra*, *Dialister pneumosintes*, *Slackia exigua*, and *Peptostreptococcus stomatis*, which form a progressively stronger co-occurrence network with disease progression^8^. Sung et al. discovered that 1 year after the eradication of HP, a distinct cluster of oral bacteria, mainly consisting of *Peptostreptococcus* and *Streptococcus*, was associated with the development and sustained presence of atrophy and intestinal metaplasia, indicating the significant role of the non-HP gastric microbiota in the progression and persistence of precancerous lesions in the stomach^9^. Another study confirmed that, in comparison to patients with chronic gastritis, GC patients exhibit a reduction in microbial diversity, a decrease in HP abundance, and concurrent enrichment of *Proteobacteria* taxa, including the genera *Phyllobacterium* and *Achromobacter* and the families *Xanthomonadaceae* and *Enterobacteriaceae*^10^. From these findings, it is evident that a comprehensive assessment of the gastric microbiota in GC is necessary to elucidate its role in the occurrence and development of GC.

In this study, we initially conducted 16S ribosomal RNA (16S rRNA) gene metabarcoding of GC tumour tissues and their corresponding normal adjacent tissues to comprehensively analyse the microbiota within GC tumours and perform microbe-based molecular subtyping. Simultaneously, transcriptomic and metabolomic analyses were performed to investigate the intricate relationship between the microbiota and host transcriptional regulation, elucidating the role of the microbiota in orchestrating metabolic reshaping within the tumour immune microenvironment (TIME). Importantly, *Streptococcus anginosus* (SA) was found to play a crucial role in the initiation and progression of GC. And SA exerts its effects by modulating the arginine metabolism pathway and regulating the TIME.

## Results

### Characterizing novel subtypes of GC based on the intratumour microbiome

**Fig. 1:**
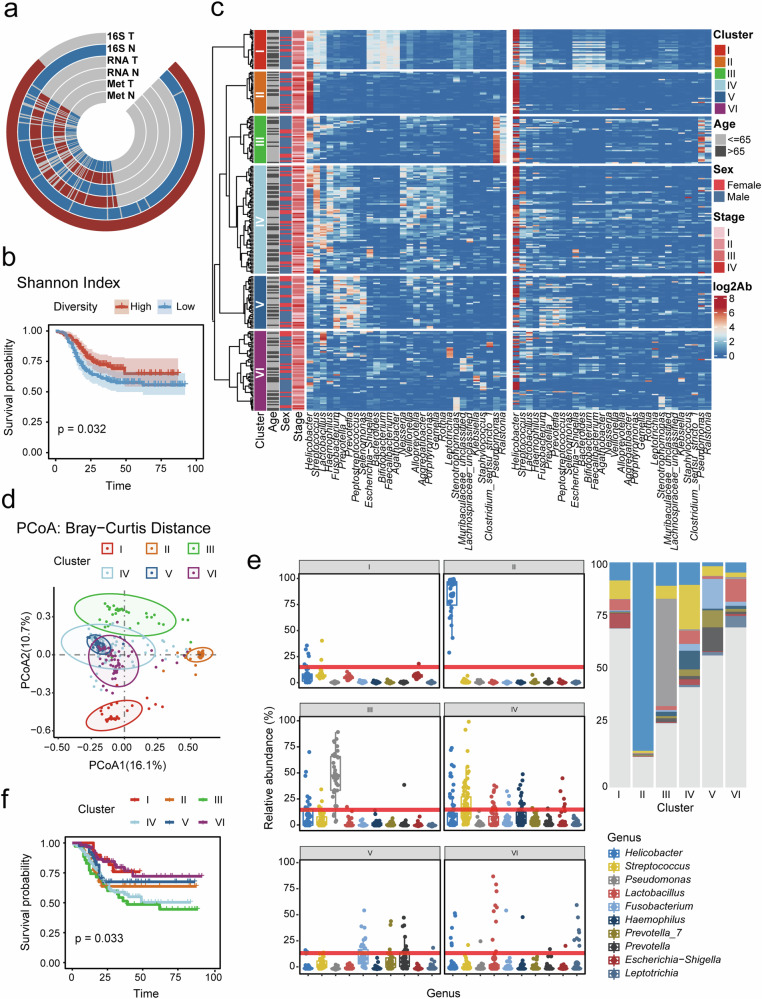
Characterizing novel molecular types of GC on the basis of the intratumoral microbiome. **a** Summary of the samples used in this multiomics study. **b** OS analysis demonstrating a significantly worse prognosis in the low-diversity group than in the high-diversity group. Alpha diversity was calculated using the *Shannon* diversity index. Survival analysis was performed using the Kaplan‒Meier method. **c** Unsupervised hierarchical clustering of tumour samples based on the 30 most differentially abundant bacterial genera between tumour and paired normal tissue samples. **d** Beta diversity patterns visualized on a principal coordinates analysis (PCoA) plot, explaining 16.1% (PCoA1) and 10.7% (PCoA2) of the total variance. Samples were clustered by tumour microbiome profile. **e** Distribution of genus-level phylotypes across the 6 identified gastric tumour microbiome clusters. **f** OS analysis indicating a significantly worse prognosis in patients in Clusters III and IV. Survival analysis was performed using the Kaplan‒Meier method.

We collected 609 samples from GC patients for multiomics analysis: (1) bacterial 16S rRNA gene metabarcoding was performed for 290 tumour (T) samples and 319 tumour-adjacent normal (N) tissues, among which 274 were paired; (2) transcriptome data were collected from 108 paired T and N samples; and (3) metabolome data were collected from 90 paired T and N samples (Fig. 1a). Patients in this cohort ranged from 28–88 years old; the cohort included 232 males and 103 females, 52 patients with stage I disease, 78 patients with stage II disease, 165 patients with stage III disease, and 40 patients with stage IV disease. More detailed clinical information on the individual patients, including age, sex, smoking and drinking status, grade of differentiation, tumour size, tumour-node-metastasis (TNM) stage, survival status and survival time, is provided in Supplementary Tables S1–S2. Pathological staging was based on the eighth edition of the American Joint Committee on Cancer’s Staging System. 16S rRNA gene metabarcoding revealed the presence of bacterial reads in all the sequenced samples, and the profiles displayed sample-specific microbiome heterogeneity (Supplementary Fig. S1a and Table S3). Among the identified bacterial taxa, *Helicobacter* was the most abundant genus in terms of overall abundance, with considerable deviation among all the samples (Supplementary Fig. S1a). To gain insights into the composition of the tumour microbiome in GC, we computed alpha diversity using the *Shannon* and *Simpson* indices. These metrics revealed a significant association between higher alpha diversity in the tumour microbiome and improved patient prognosis (Fig. 1b; Supplementary Fig. S1b), which was consistent with the findings of previous studies^11,12^.

The high heterogeneity of the tumour microbiome highlights the potential existence of novel subgroups within the GC patient cohort. We then clustered tumour samples into six groups based on the abundance of the top 30 genera using the unsupervised clustering method^13^ (Fig. 1c; Supplementary Table S4). Notably, the bacterium-based clusters were not correlated with patient age, sex, or cancer stage (Fig. 1c; Supplementary Fig. S1c). Furthermore, we employed the Bray‒Curtis distance^14^ to calculate beta diversity, which successfully distinguished these clusters on PCoA1 and PCoA2 (Fig. 1d). And we calculated the Euclidean distance (Eu-dist) on CLR-transformed data and compared the result with the former beta-diversity represented by Bray-Curtis distance (Br-dist). Both methods showed larger inter-cluster distances than intra-cluster distances (Eu-dist < 13 and Br-dist ≤ 0.8), thus distinguishing different clusters (Supplementary Fig. S1d). Each cluster was characterized by varying enrichment of particular genera. Interestingly, three clusters exhibited enrichment of genera that accounted for > 15% of the total bacteria: *Helicobacter*, *Pseudomonas* and *Streptococcus* accounted for 84.06%, 47.93% and 19.80%, respectively, of the bacteria in Cluster II, Cluster III, and Cluster IV (Fig. 1e). Clusters with enriched genera tended to have lower alpha diversities, as calculated by both the *Shannon* index and the *Simpson* index (Supplementary Fig. S1e).

To understand the clinical relevance of these newly identified clusters, we compared the overall survival (OS) of GC patients assigned to different clusters. Significantly divergent outcomes were observed among the clusters (Fig. 1f). We applied univariate and multivariate Cox regression, taking gender, age, family history, smoking, drinking, tumour location, tumour size, grade of differentiation, TNM stage, operation mode, postoperative chemotherapy and the bacterium-based clusters into account. As shown in Supplementary Table S5, both univariate and multivariate Cox regression analyses suggest that those bacterium-based clusters were independent prognostic factors for GC. Specifically, patients in Cluster III and Cluster IV had poorer overall survival than did those in the other clusters, which suggested that *Pseudomonas* and *Streptococcus* may contribute to the progression of GC (Fig. 1f). Previous research has highlighted the potential impacts of specific bacteria on carcinogenesis^10,15,16^ and cancer metastasis^17^, which led us to further explore the abundant genera within these two distinct clusters.

### Impacts of the intratumoral microbiota on oncogenic pathways and the TIME in GC

**Fig. 2:**
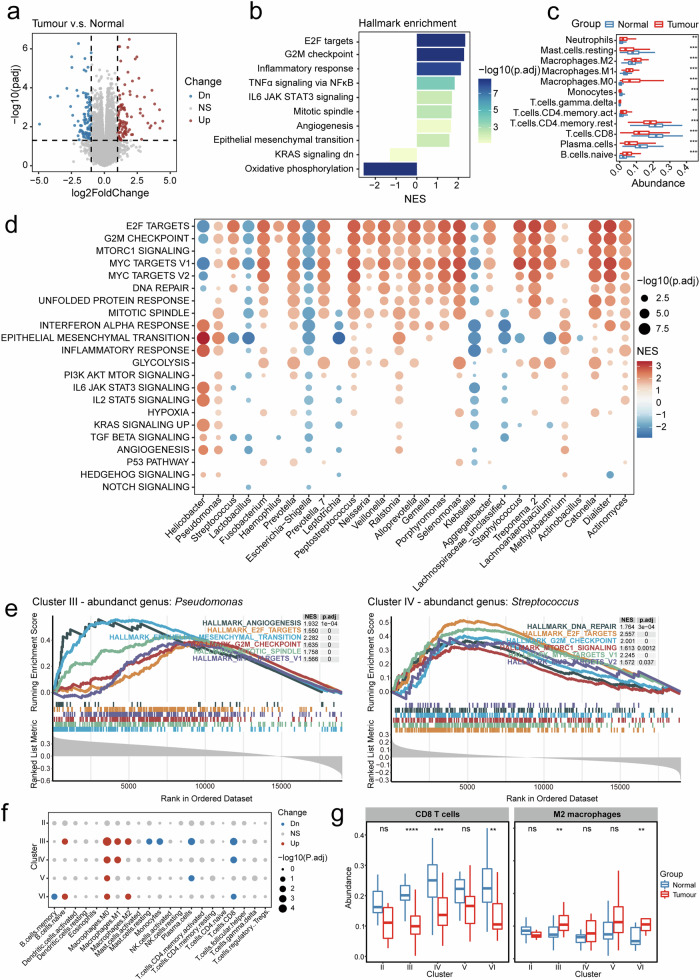
Bacteria play an important regulatory role in the TIME of GC. **a** Differential analysis demonstrating differentially expressed genes (DEGs) between tumour and normal samples. Significance thresholds: adjusted *p*-value < 0.05 and fold change > 2. **b** Gene set enrichment analysis (GSEA) results for hallmark pathways enriched in DEGs from (**a**). Significance thresholds: adjusted *p*-value < 0.05 and | NES | > 1. **c** Predicted immune cell infiltration levels in tumour versus normal samples calculated by CIBERSORT. Significance threshold: adjusted *p*-value < 0.05. **d** GSEA results for hallmark pathways enriched among genes correlated with the 30 most abundant bacterial genera. Significance thresholds: adjusted *p*-value < 0.05 and | NES | > 1. **e** Examples of GSEA hallmark pathway enrichment for genes correlated with *Pseudomonas* abundance (left) and *Streptococcus* abundance (right). **f** Predicted immune infiltration in the 5 tumour microbiome clusters. Significance threshold: adjusted *p*-value < 0.05. **g** Infiltration levels of CD8^+^ T cells and M2 macrophages in normal and tumour samples across clusters.

To elucidate the gene expression landscape, we conducted RNA sequencing (RNA-seq) analysis of 108 tumour tissues and paired normal tissues. Differential expression analysis revealed 106 upregulated and 91 downregulated genes in tumour samples compared to normal samples (Fig. 2a; Supplementary Table S6). The hallmark terms associated with protumoral processes, including E2F targets, the G2M checkpoint, and epithelial–mesenchymal transition, were positively enriched in the tumour samples according to gene set enrichment analysis (GSEA) (Fig. 2b). Conversely, oxidative phosphorylation, a key metabolic pathway that is often dysregulated in cancer according to the Warburg theory^18^, was negatively enriched in GC tumours (Fig. 2b).

Moreover, we utilized CIBERSORT^19^ to predict the immune infiltration patterns of both tumour and normal tissues based on expression profile data. Our findings revealed a reduced abundance of antitumoral immune cells, such as CD8^+^ T cells and plasma cells, in tumour samples. Conversely, M2 macrophages, which can promote immune suppression, angiogenesis, and neovascularization, had an increased abundance in tumour samples (Fig. 2c; Supplementary Fig. S2a).

We further investigated the differences in gene expression across the aforementioned clusters and detected distinct differentially expressed genes (DEGs) between tumour tissues and paired normal tissues within these clusters; clusters III and V had more DEGs than did the other clusters (Supplementary Fig. S2b). Notably, clusters III and IV also exhibited cluster-specific DEGs compared to the other clusters, suggesting that the bacteria enriched in these clusters may be correlated with dynamic changes in the transcriptome (Supplementary Fig. S2c). To understand how bacteria may contribute to these patterns, we calculated the statistical correlations between the abundances of bacterial genera and gene expression^19^. The genes significantly correlated with the 30 most abundant genera in tumour samples were enriched in diverse hallmark pathways. Notably, cell cycle-related pathways, including E2F targets and the G2M checkpoint, were among the most enriched genus-associated pathways (Fig. 2d). Furthermore, differences were observed in the enrichment patterns of genus-correlated genes. For instance, genes significantly correlated with the Cluster III-abundant genus *Pseudomonas* and the Cluster IV-abundant genus *Streptococcus* displayed positive enrichment in protumorigenic pathways such as angiogenesis, mechanistic target of rapamycin complex 1 (mTORC1) signalling, MYC targets, and DNA repair (Fig. 2d–e). In contrast, genes correlated with *Lactobacillus*, which is widely recognized as a probiotic in the human gastrointestinal tract^20,21^, were negatively enriched in these protumoral pathways (Fig. 2d).

Furthermore, we also used CIBERSORT to predict immune infiltration patterns in Clusters III and IV and observed differences in the immune infiltration landscape within these clusters. Both clusters showed depleted antitumour CD8^+^ T cells but elevated numbers of tolerant/evasive M2 macrophages in tumours (Fig. 2f–g). These findings align with the observed poorer overall survival outcomes (Fig. 1f) and imply the potential influence of abundant bacteria in these clusters on the tumour microenvironment and immune responses.

### SA is a risk factor correlated with tumorigenesis and reduced infiltration of CD8^+^ T cells in GC

**Fig. 3:**
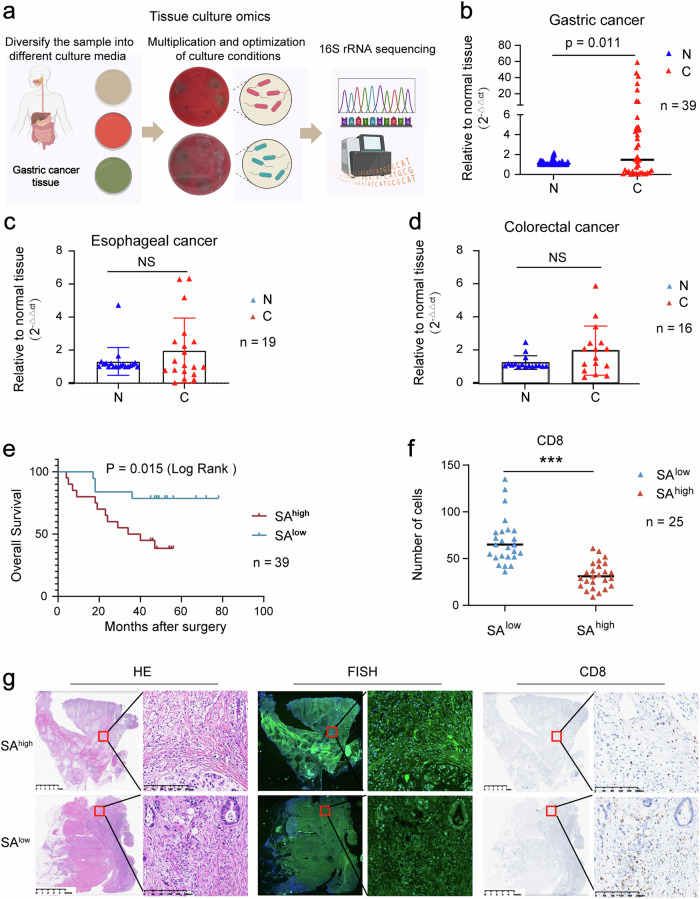
SA is a prognostic risk factor for GC and is negatively correlated with CD8^+^ T lymphocyte counts. **a** We cultured cancer tissues from 10 patients within specific clusters of interest (i.e., Clusters III and IV) to identify key microbes affecting the occurrence and development of GC. **b** qPCR was used to detect the differential abundance of SA between GC tissues and corresponding normal tissues. **c** qPCR was used to detect the differential abundance of SA between oesophageal cancer tissues and corresponding normal tissues. **d** qPCR was used to detect the differential abundance of SA between colorectal cancer tissues and corresponding normal tissues. **e** Kaplan–Meier analysis of survival differences between GC patients with high expression vs low abundance of SA. **f** Correlation analysis between the abundance of SA and that of CD8^+^ T lymphocytes. **g** Representative images of H&E staining of tumour tissues, FISH detection of SA, and immunohistochemical detection of CD8^+^ T lymphocytes in tumour tissues.

To further clarify the role of the microbiota in the occurrence and development of GC, we cultured cancer tissues from 10 patients within specific clusters of interest (i.e., clusters III and IV), isolating and identifying five species of bacteria: *Streptococcus anginosus* (SA), *Streptococcus halitosis*, *Bacillus koreensis*, *Listeria monocytogenes*, and *Burkholderia* sp. (Fig. 3a). We calculated the ANI values of the clinical isolate *Streptococcus anginosus_ZJCC* (This is the strain of bacteria we isolated from patient tissue) with several reference strains of this species, and all ANI values were > 95%, indicating that our isolated and functionally validated strain is indeed SA (Supplementary Fig. S3). Previous studies have shown that the abundance of SA in the microbiota is significantly greater in GC tissues than in normal tissues^22^. We investigated the abundance and clinical significance of SA in GC tissues in another independent cohort of 39 GC patients. Similarly, we found that SA was significantly more abundant in GC tissues than in normal tissues (Fig. 3b). Moreover, to assess whether SA is specifically present at high levels in GC tissues, we also collected samples from oesophageal and colorectal cancers, in which SA was not present at higher levels in cancer tissues than in adjacent normal tissues (Fig. 3c, d). Additionally, both univariate and multivariate Cox regression analyses suggest that the abundance of SA was independent prognostic factors for GC (Fig. 3e; Supplementary Table S7).

In addition, to clarify the regulatory role of SA in the TIME of GC, we used immunohistochemistry and fluorescence in situ hybridization (FISH) to investigate the correlation between bacterial populations and the CD8^+^ T lymphocyte population. We observed a significant reduction in CD8^+^ T cells in the group with higher SA abundance (Fig. 3f–g).

### SA promoted GC initiation and growth by reshaping the TIME in vivo

**Fig. 4:**
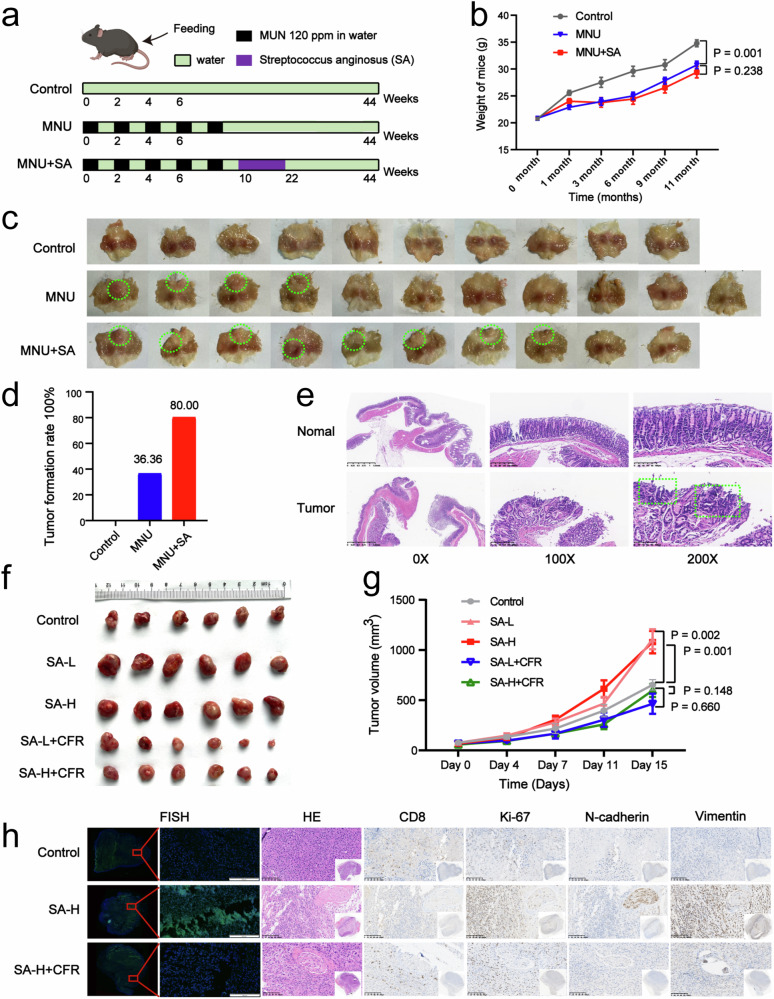
SA regulates the TIME to promote GC occurrence and growth in mice in vivo. **a** Establishment and treatment of the N-methylnitrosourea (MNU)-induced spontaneous GC model. **b** Body weight of mice in the MNU-induced spontaneous GC model. **c** Representative images of mouse gastric mucosal tumour formation. **d** Differences in tumour incidence in the MNU-induced spontaneous GC model. **e** H&E staining revealed that the polypoid mass was an intramucosal adenocarcinoma. **f** Image showing the tumour sizes in each group in the mouse xenograft experiment. **g** Tumour volumes in each group in the mouse xenograft experiment. **h** Representative images of H&E staining of tumour tissues, FISH detection of SA, and immunohistochemical detection of CD8^+^ T lymphocytes, Ki-67, N-cadherin and vimentin in tumour tissues. SA-L meant low-dose SA, SA-H meant high-dose SA, SA-L + CFR meant low-dose SA group treated with ceftriaxone (CFR), SA-H + CFR meant high-dose SA group treated with ceftriaxone (CFR).

We used an N-methylnitrosourea (MNU)-induced spontaneous GC model to observe the effect of SA on the occurrence of GC (Fig. 4a). Compared with those in the control group, the weights of the mice in the MNU model group and the MNU combined with SA intervention group were significantly lower (Fig. 4b). In addition, the GC tumour formation rate in the MNU model group was 36.36%, while that in the MNU combined with SA intervention group was 80%, which was more than two times greater than that in the MNU model group (Fig. 4c–d). Haematoxylin-eosin staining revealed the macroscopic and microscopic appearance of the GCs, with specific masses indicating intratumoral adenocarcinoma (Fig. 4e). Intervention with SA can promote the development of GC, and both MNU and SA can cause gastric mucosal lesions in mice, thereby affecting their nutritional status and ultimately leading to weight loss.

In addition, we used a mouse transplanted tumour model to observe the effect of SA on the proliferation, invasion, and metastasis of GC. We used mouse-derived MFC cells to construct a mouse transplanted tumour model and administered low and high doses of SA. The results showed that both low and high doses of SA promoted tumour proliferation, and the tumour volume significantly increased in the SA-treated groups compared to that in the control group (Fig. 4f–g). Moreover, after antibiotic intervention, these effects were reversed (Fig. 4f–g). Considering that SA and Streptococcus halitosis were simultaneously cultured from GC tissues, we further explored whether both of them could promote the growth of GC. Further experiments have shown that SA can promote the growth of GC, while *Streptococcus halitosis* cannot in vivo (Supplementary Fig. S4a–d).

Furthermore, through FISH, we observed that the intratumoral SA concentration increased significantly after SA intervention, while most SA was eliminated after antibiotic intervention. The immunohistochemical results indicated that CD8^+^ T lymphocyte numbers were significantly reduced in the SA intervention group. The expression levels of Ki-67, which indicates proliferation, N-cadherin and vimentin, which indicate invasion and metastasis, were significantly increased; these phenomena were reversed after antibiotic intervention (Fig. 4h). These results suggested that SA may simultaneously regulate CD8^+^ T lymphocytes and tumour cells in the TIME, thereby promoting the occurrence and development of GC.

### SA promoted cancer development and inhibited CD8^+^ T-cell differentiation in vitro

**Fig. 5:**
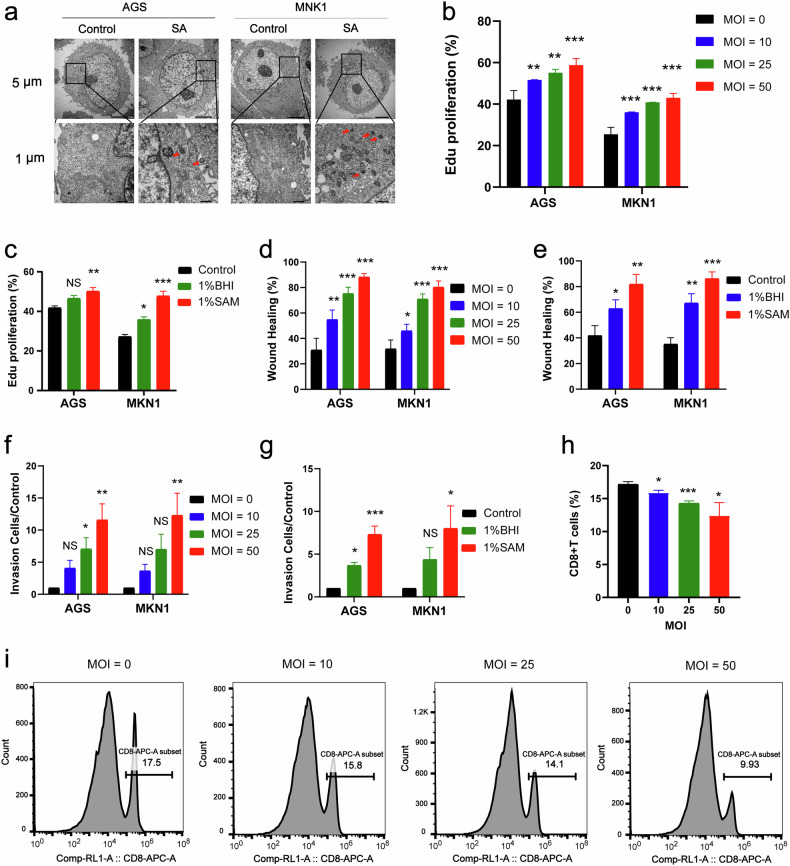
SA promotes the proliferation, migration and invasion of GC cells while inhibiting the differentiation of CD8^+^ T cells in vitro. **a** SA-infected cells were detected by transmission electron microscopy, and the red arrows indicate SA. **b**, **c** The proliferation ability of GC cells with or without SA infection (**b**) or treatment with metabolites of SA (**c**) was examined via an EdU incorporation assay. **d**, **e** The migration ability of GC cells with or without SA infection (**d**) or treatment with metabolites of SA (**e**) was examined via wound healing experiments. **f**, **g** The invasion ability of GC cells with or without SA infection (**f**) or treatment with metabolites of SA (**g**) was examined by transwell invasion experiments. **h** Differentiation of CD8^+^ T cells infected with SA was examined via flow cytometry. **i** Representative flow cytometry plots. All the experiments were repeated 3 times. *p < 0.05; **p < 0.01; ***p < 0.001.

To investigate the possibility that SA invades live GC cells, we conducted a coculture experiment involving the GC cell lines AGS and MKN1. The cells were exposed to SA at an MOI of 10 for 2 h and subsequently subjected to transmission electron microscopy (TEM) analysis. Our TEM findings unequivocally revealed the invasion of both AGS and MKN1 cells by SA (Fig. 5a).

Because the metabolites generated through microbial metabolic processes are pivotal for mediating interactions between microbial communities and host organisms^23^, we cocultivated SA and its metabolites separately with GC cells to observe their effects on the proliferation, migration, and invasion abilities of these cells. 5-Ethynyl-2-deoxyuridine (EDU) experiments indicated that in culture with GC cells, both SA (Fig. 5b; Supplementary Fig. S5a) and its metabolites (Fig. 5c; Supplementary Fig. S5b) significantly enhanced cell proliferation. Furthermore, wound healing experiments also indicated that cocultivation of SA (Fig. 5d; Supplementary Fig. S5c) and its metabolites (Fig. 5e; Supplementary Fig. S5d) with GC cells significantly enhanced the healing and migration capabilities of these cancer cells. Moreover, Transwell invasion experiments demonstrated that both SA (Fig. 5f; Supplementary Fig. S5e) and its metabolites (Fig. 5g; Supplementary Fig. S5f) promoted the invasion ability of GC cells.

In addition, we observed the regulation of CD8^+^ T lymphocytes in the TIME by SA in vivo. To investigate the differentiation of CD8^+^ T lymphocytes, we collected peripheral blood mononuclear cells (PBMCs) from healthy individuals and cocultured them with SA and its metabolites. The results indicated that SA could inhibit the differentiation of CD8^+^ T lymphocytes in human PBMCs (Fig. 5h–i). In conclusion, SA and its metabolites can promote the proliferation, migration, and invasion of GC cells. Moreover, SA can inhibit the differentiation of CD8^+^ T lymphocytes, leading to immune evasion by tumour cells, which may further promote tumour growth.

### A high abundance of Streptococcus enhances the expression of components of arginine metabolic pathways in tumour samples

Previous research has revealed the associations between clinical features and metabolic functions performed by bacteria^24^. Specific bacterium-derived metabolites, such as inosine, have also been shown to modulate the response to checkpoint inhibitor immunotherapy^25^. To elucidate the mechanisms underlying the potential influence of bacteria on tumorigenesis and progression, we analyzed the metabolomes of 90 tumour samples and paired normal tissues. A total of 778 unique metabolites with clear kyoto encyclopaedia of genes and genomes (KEGG) annotations were categorized into eight main metabolic groups: carbohydrate metabolism, lipid metabolism, nucleotide metabolism, amino acid metabolism, cofactors and vitamin metabolism, xenobiotic biodegradation, terpenoid and polyketide metabolism, and signal transduction (Supplementary Fig. S6a and Table S8).

**Fig. 6:**
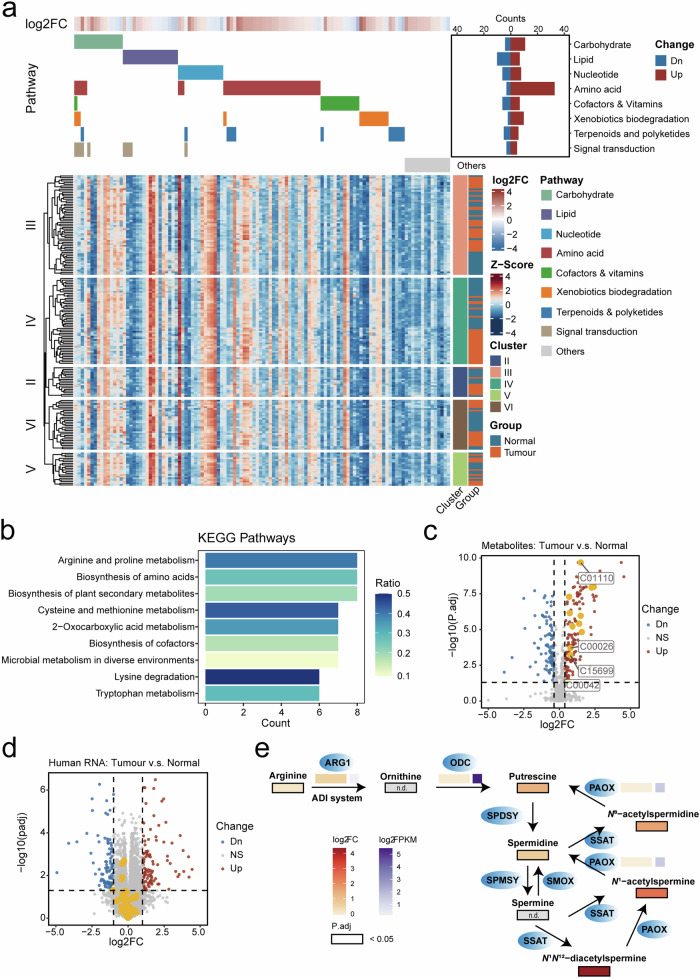
Metabolomic analysis revealed increased arginine metabolic activity in tumour samples, particularly in Cluster IV tumour samples enriched with *Streptococcus.* **a** Metabolite landscape representing differentially abundant metabolites within different clusters annotated by metabolite-related pathways. The numbers of differentially abundant metabolites in each pathway were displayed (top right). Significance thresholds: adjusted *p*-value < 0.05 and fold change > 1.3. **b** Specific KEGG pathways enriched within the amino acid metabolism group. **c** Differential abundance analysis demonstrating differentially abundant metabolites between tumour and normal samples, with metabolites involved in arginine and proline metabolism were highlighted in yellow. Significance thresholds: adjusted *p*-value < 0.05 and fold change > 1.3. **d** Differential expression analysis of DEGs between tumour and normal samples; genes involved in arginine and proline metabolism were highlighted in yellow. Significance thresholds: adjusted *p*-value < 0.05 and fold change > 2. **e** Schematic representation of arginine metabolic pathways in bacteria. Fold changes between tumour and normal tissues were colored white to red; expression levels were colored white to purple.

Overall, we identified 148 significantly upregulated metabolites and 93 downregulated metabolites. Notably, these differentially abundant metabolites had different proportions across metabolic groups, and amino acid metabolism exhibited the largest number of differentially abundant metabolites, especially those upregulated in tumour samples (Fig. 6a; Supplementary Table S9). Specifically, tumour and normal tissues could be well separated in Cluster IV based on metabolite abundance, with larger metabolic alterations in Cluster IV GC samples (Fig. 6a; Supplementary Fig. S6a–b).

To identify pathways influenced by specific bacteria, we calculated the numbers and ratios of KEGG pathways associated with amino acid metabolism. Arginine and proline metabolism was the most enriched pathway (Fig. 6b). All metabolites related to arginine metabolism were consistently upregulated in tumour samples (Fig. 6c), while human genes encoding factors involved in this pathway showed no significant change at the transcriptomic level (Fig. 6d). This discrepancy between the overall metabolome and the human-specific transcriptome for arginine metabolism suggests a potential bacterial role in modulating this metabolic process. Indeed, bacteria, including *Streptococcus* spp., are capable of metabolizing arginine. The metabolites downstream of this pathway all exhibited significant increases in abundance, such as *N*^1^-acetylspermine (6.02-fold higher) and *N*^1^*N*^12^-diacetylspermine (20.37-fold higher), in tumour tissues compared to normal tissues. In contrast, the expression of enzymes related to this pathway in the human transcriptome did not change (Fig. 6e). In conclusion, arginine metabolism was enriched in *Streptococcus*-abundant GC samples without significant changes in human arginine enzymes, suggesting that the potential role of *Streptococcus* in GC may involve bacterial arginine metabolism.

### SA metabolizes arginine into ornithine to promote tumour growth

**Fig. 7:**
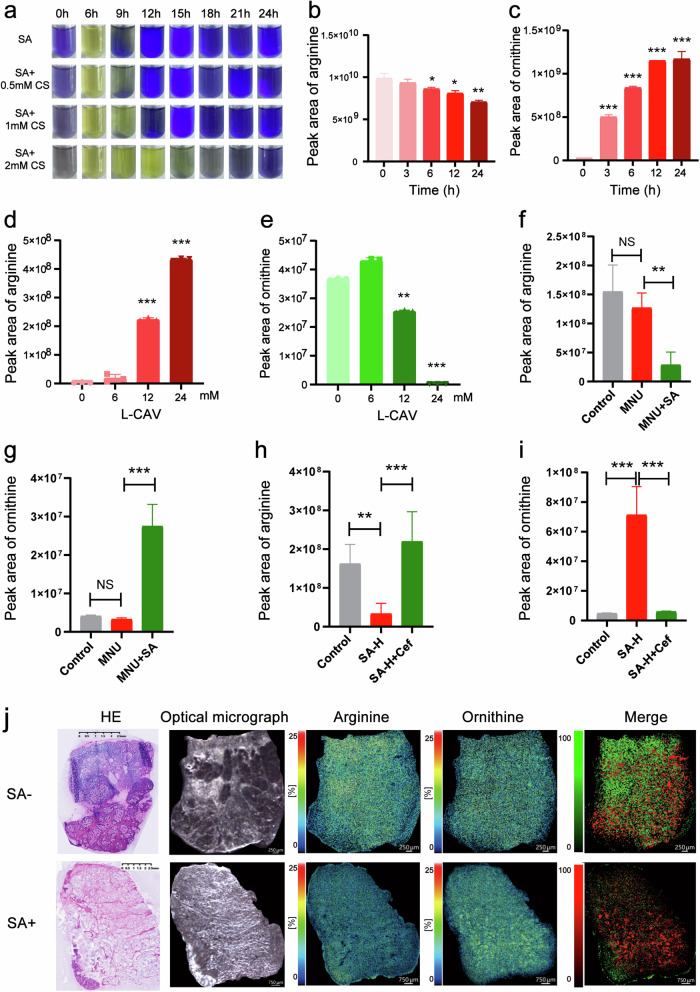
SA induces tumour growth by metabolizing arginine into ornithine to regulate the TIME. **a** Bacterial arginine dihydrolase was used to observe the discolouration process caused by arginine metabolism in SA treated with different concentrations of arginine deaminase (ADI) inhibitors (L-canavanine sulfate, L-CAV), and the results showed that L-CAV can inhibit SA arginine metabolism in a concentration-dependent manner. **b**, **c** Mass spectrometry to determine the levels of arginine (**b**) and ornithine (**c**) at different time points (0 h, 3 h, 6 h, 12 h, and 24 h) after coculturing SA with arginine. **d**, **e** Mass spectrometry was used to measure the levels of arginine (**d**) and ornithine (**e**) after coculturing SA with arginine and with different concentrations of the ADI inhibitor L-CAV (0 mM, 6 mM, 12 mM, or 24 mM). **f**, **g** Changes in the levels of arginine (**f**) and ornithine (**g**) in various groups of tumour tissues in the MNU-induced spontaneous GC model. **h**, **i** Levels of arginine (**h**) and ornithine (**i**) in various groups of tumour tissues in the MFC cell xenograft model experiment. **j** Mass spectrometry was used to determine the levels of arginine and ornithine in tumour tissues from SA-positive and SA-negative patients, with green representing arginine and red representing ornithine in the merged image. All experiments were repeated 3 times. *p < 0.05; **p < 0.01; ***p < 0.001.

Metabolomic analysis revealed that all metabolites related to arginine metabolism are consistently upregulated in tumour samples, and the microbiome plays a crucial role in this process, particularly in Cluster IV tumour samples enriched with *Streptococcus*. Numerous previous studies have shown that arginine deaminase (ADI) produced by the microbial community can catalyse the conversion of arginine to ornithine, which plays a crucial regulatory role in the TIME and ultimately leads to immune evasion and tumour progression^26–28^. Therefore, we introduced SA and the ADI inhibitor L-canavanine sulfate (L-CAV) into arginase dihydrolase broth to evaluate the ability of SA to metabolize arginine and observed colour changes at 0, 6, 12, and 24 h (Fig. 7a). Over time, we observed a continuous decrease in arginine levels and a gradual increase in ornithine levels, indicating that SA can metabolize arginine to produce ornithine (Fig. 7b, c; Supplementary Fig. S7a–d). L-CAV reversed this process in a concentration-dependent manner (Fig. 7d, e; Supplementary Fig. S7e, f).

In addition, in the MNU-induced spontaneous GC mouse model, nontargeted metabolomic sequencing was performed on tumour tissues from each group. Compared with those in the control group and the MNU model group, the level of arginine was significantly lower and the level of ornithine was greater after the SA intervention (Fig. 7f–g). In the mouse xenograft tumour model, we also observed a significant decrease in arginine levels and an increase in ornithine levels in the SA intervention group compared to the control group. In addition, these changes were reversed after antibiotic intervention (Fig. 7h–i). Furthermore, we intervened with arginine and ornithine, as well as their downstream metabolites N1 acetylspermine and N8 acetylspermine, in a GC xenograft model and found that they can promote the growth of GC. This suggests that activation of arginine and its downstream pathways can promote the growth of GC, and SA may play a role in promoting GC growth by metabolizing arginine to produce ornithine and activating its downstream pathways (Supplementary Fig. S8a–d).

Finally, at the clinical level, we utilized mass spectrometry imaging to observe the spatial expression of arginine and ornithine in the SA-positive and SA-negative groups. The results indicated that compared to SA-negative patients, those in the SA-positive group exhibited a significant decrease in arginine levels and an increase in ornithine levels within tumour tissues (Fig. 7j). Given that the differentiation of CD8^+^ T lymphocytes was inhibited in PBMCs, SA’s simultaneous regulation towards tumour cells and CD8^+^ T lymphocytes in the TIME might influenced by its arginine metabolic pathway, which could ultimately promote the occurrence and progression of GC.

## Discussion

Due to the harsh acidic conditions of the stomach and the limitations of previously used culture techniques, studying the gastric microbiota has posed significant challenges to researchers. However, with the advent of new PCR techniques and metagenomic analysis, an increasingly diverse range of microbial species are being discovered within gastric tissues^29,30^. In our study, for the first time, we classified GCs into six clusters based on the tumour-resident microbiota, with each cluster characterized by different degrees of enrichment of specific genera. Interestingly, patients in cluster III (47.93% *Pseudomonas*) cluster IV (19.80% *Streptococcus*) had the worst prognosis. This finding suggested that in addition to HP, *Pseudomonas* and *Streptococcus* may also play significant roles in the onset and progression of GC.

Furthermore, we cultured and detected SA in GC tissues and confirmed its significantly greater abundance in GC tissues than in normal tissues and its association with poor prognosis. SA, a common gram bacterium, is widely distributed in the environment; in human oral, nasal, and pharyngeal sites; and in the gastrointestinal tract^31–33^. Previous studies have indicated a noticeable increase in SA abundance in the oral cavity, mucosa, and faeces of GC patients, which makes SA a potential biomarker for GC diagnosis^8,34^. Additionally, SA has been proven to be closely associated with the occurrence of GC and liver metastasis^35^. Another study showed that *Streptococcus* is the second most common bacterium in HP-positive GC patients and accounts for the highest proportion of bacteria in the HP-negative GC microbiome^36,37^. After the eradication of HP, *Streptococcus* abundance significantly increases in patients with sustained inflammation and intestinal metaplasia^9^, indicating that it SA may play a crucial role in GC occurrence and development in addition to HP. Surprisingly, we observed the promotion of GC by SA in spontaneous GC mouse models and transplanted tumour GC mouse models, as well as enhanced proliferation and invasion and migration capacity of GC after coculture with SA in vitro.

The intratumoral microbiota constitutes an essential component of the TIME^38^, and tumour-resident bacteria in the TIME may be involved in cancer onset and development through various immune mechanisms and metabolic remodelling pathways^39^. Previous studies have shown that microbiome–immune networks within the breast tend to be bacterium-centric, with sparse structures in tumours and more interconnected structures in benign tissues^40^. In addition, *Propionibacterium* and *Staphylococcus*, which are depleted in tumours, are negatively associated with oncogenic immune features and are significantly correlated with T-cell activation-related genes^40^. In oesophageal cancer, *Streptococcus* can induce a favourable chemotherapeutic immune response by stimulating CD8^+^ T-cell infiltration^41^. In oral squamous cell carcinoma, *Streptococcus*-reactive cytotoxic CD8^+^ T cell numbers are significantly decreased in advanced-stage patients compared to early-stage patients and healthy individuals^42^. In our study, transcriptomic analysis revealed a significant decrease in CD8^+^ T cell numbers within the cancer tissues of Cluster IV patients characterized by high levels of *Streptococcus*. Our in vivo animal experiments further confirmed that SA can reduce the infiltration of CD8^+^ T lymphocytes into transplanted tumours. Additionally, in vitro experiments showed that SA can inhibit the differentiation of CD8^+^ T lymphocytes in PBMCs obtained from healthy volunteers.

Numerous studies have indicated that the microbiota can establish an immune-microbe-metabolism axis with the host by altering metabolic pathways, thereby regulating host immune metabolism and function and influencing the initiation and progression of tumours^43,44^. We identified arginine metabolism as the most enriched pathway through metabolomic analysis of GC tissues. All metabolites associated with arginine metabolism were consistently upregulated in tumour samples, while human-encoded genes involved in this pathway did not exhibit significant changes at the transcriptomic level. This finding suggests that the microbiota play a crucial role in the arginine metabolism pathway in GC. Arginine, a semiessential amino acid, plays crucial physiological roles in the body and is involved in three primary processes: the urea cycle, nitric oxide synthesis, and polyamine metabolism^45^. It contributes to the metabolic regulation of the tumour immune microenvironment, including the proliferation, survival, and protein synthesis of cancer and immune cells^46^. The functionality of CD8^+^ T cells relies on arginine intake, and the proliferation, survival capacity, memory formation, and antitumour efficacy of these cells are enhanced by arginine^47^. Arginine metabolism primarily depends on the activity of the nitric oxide synthase (NOS) and arginase (ARG) families^48^.

In bacteria, the ADI pathway is the most widespread pathway for arginine degradation and converts L-arginine into L-ornithine, ammonia, and carbon dioxide^49–51^. Several studies have shown that streptococcal ADI can inhibit the activation and differentiation of CD4^+^ T and CD8^+^ T cells in human PBMCs. Our research revealed that ADI inhibitors suppress the production of ornithine from arginine in a concentration-dependent manner. Additionally, spatial metabolomics revealed that GC tissues from patients in the SA-positive group exhibited decreased levels of arginine and increased levels of ornithine, while those from patients in the SA-negative group exhibited the opposite trend. Therefore, SA regulates the function of tumour cells and CD8^+^ T cells in the TIME through the arginine metabolism pathway, thereby promoting the initiation and progression of GC.

Overall, we conducted molecular subtyping of GC based on the intratumoral microbiota for the first time and revealed that different microbial characteristics have diverse prognostic and clinical implications. Furthermore, we discovered and confirmed that, in addition to HP, SA plays a crucial role in the initiation and progression of GC. Finally, our findings confirmed that SA regulates the proliferation, migration, and invasion of tumour cells while also inhibiting the differentiation and infiltration of CD8^+^ T lymphocytes in the TIME by altering the arginine metabolic pathway, ultimately promoting the onset and progression of GC. Therefore, SA may serve as a promising therapeutic target for GC treatment.

## Materials and methods

### Collection and preparation of clinical specimens

This study included samples obtained from 335 patients (290 tumour (T) samples and 319 tumour-adjacent normal (N) tissue samples, among which 274 were paired) of the Zhejiang Cancer Hospital from January 2013 to December 2018. The Research Ethics Committees of Zhejiang Cancer Hospital approved the study (No. IRB-2023-791), and all patients provided written informed consent. The informed consent form clearly informed the patients that all clinical information, such as age, sex, and TNM staging, would be used for academic research and publications. These patients were all newly diagnosed patients with GC who underwent surgical resection and had received no prior treatment for this disease, including chemotherapy, radiotherapy, targeted therapy, or biological therapy. Patients who were found to have two or more malignancies were excluded.

Tumour tissues and paired normal tissue samples were collected from the same patients at the time of surgical resection. Notably, normal tissue samples were collected from regions within ~2 cm of the corresponding tumour sites. The sample size was approximately 0.5 × 0.5 cm, and four to five tumour specimens and normal tissue samples were collected for most patients. For metabolomics analyses, the tissue specimens were subjected to cold ischaemia for < 30 min prior to freezing a –80 °C freezer. For 16S rRNA gene metabarcoding and transcriptomic analyses, tissue specimens were soaked in RNA protective solution (Cat No. RO901-500ML, Sigma-Aldrich, Massachusetts, America) at 4 °C overnight and then frozen in a –80 °C freezer. Histologic sections obtained from the top and bottom portions of each specimen were reviewed by a senior board-certified pathologist to confirm that the tissues were tumours or normal tissues. The top and bottom sections of tumour samples had to contain an average of 60% tumour cell nuclei with < 20% necrosis to be deemed acceptable for this study.

### 16S rRNA gene metabarcoding

Microbial DNA was extracted using an E.Z.N.A. Tissue DNA Kit (D3396-01; Omega, Norcross, Georgia, USA) following the manufacturer’s instructions. The DNA was quantified using a Qubit 2.0 fluorometer (Invitrogen, Carlsbad, CA, USA), and the molecular size was estimated via agarose gel electrophoresis. Primers targeting the hypervariable V3-V4 region of the 16S rRNA gene were used to amplify the extracted DNA samples. The methods and analytical steps are detailed in the Supplementary Data S1.

### RNA-seq assays

mRNA sequencing (RNA-seq) was performed on paired tumour tissue and normal tissue samples from 108 AEG patients. For mRNA-seq, a library was prepared from 1 μg of DNase I-treated total RNA using a TruSeq kit (Illumina), and 150-bp paired-end sequencing was performed on an Illumina HiSeq X Ten machine at LC-Bio Technology Co., Ltd. (Hangzhou, Zhejiang, China), following the vendor’s recommended protocol. The methods and analytical steps are detailed in the Supplementary Data S1.

### Metabolomic assays

The samples were subjected to careful deproteinization preprocessing before mass spectrometry analysis. Quality control samples were inserted during the analysis to assess technical reproducibility. The raw data were converted using MSConvert, peak extraction was performed with XCMS, adduct and ion annotation was conducted using CAMERA, and final identification was done with metaX, using a library derived from an in-house standard compound database. Differentially abundant metabolites were identified by the Wilcoxon test. Metabolites with a FC ≥ 1.3 and an adjusted *p*-value < 0.05 were considered to be significantly different. The methods and analytical steps are detailed in the Supplementary Data S1.

### Culturomics-based isolation of single bacteria from GC tissue

Gastric cancer tissues were ground and subjected to multiple gradient dilutions, and then the diluted tissue homogenates were applied to Columbia Blood plates. The plates were incubated at 37 °C for 48 h and individual clones were selected. The single clones were amplified to a certain amount and the DNA was extracted and amplified by PCR using 27 F and 1429 R as primers. The amplified products were then sent to Sangon Biotech for sequencing. The sequencing results were compared with those in the NCBI database to isolate and preserve individual bacterial species, and some of the *Streptococcus anginosus* isolated were subjected to next-generation sequencing for further strain identification. For detailed steps on the isolation of individual bacteria, please refer to the Supplementary Data S1.

### Bacterial culture and total metabolic products of SA (SAMs)

SA obtained through a GC tissue culture-based omics approach was cultured in brain heart fusion broth (BHI) (Huankai Microbial Technology Co., Ltd., Guangzhou, China) at 37 °C. After SA was cultivated to a concentration of 1 × 10^9^ CFU/mL, the bacterial culture medium was centrifuged at 12,000 rpm for 15 min, the supernatant was collected, and the mixture was passed through a 0.22 μm filter with a pore size of m to obtain the SAMs.

### Cell lines and cell culture

Human GC cell lines, including AGS and MKN1 cells, and the mouse MFC cell line were obtained from Cobioer Biosciences Co., Ltd. (Nanjing, China). These three cell lines were identified by short tandem repeats, and bacterial and fungal contamination tests were negative. For detailed culture methods, please refer to the Supplementary Data S1.

### MNU-induced spontaneous GC model

The mice were divided into three groups, each comprising 12 male C57BL/6 mice aged 4–6 weeks: the control group, the MNU group, and the MNU + SA group. Initially, GC induction was performed on 24 of the mice using MNU. All animal experiments were approved by the Institutional Animal Care and Use Committee of Zhejiang cancer hospital (2022-08-110). For detailed animal experimental procedures and analysis methods, please refer to the Supplementary Data S1.

### Transplanted tumour model with subcutaneously transplanted MFC cells

The experiment comprised 5 groups, each with 6 male 615 mice aged 6–8 weeks, for a total of 30 mice. The groups included the control group, low-dose SA group, high-dose SA group, low-dose SA group treated with ceftriaxone (CFR), and high-dose SA group treated with CFR. All animal experiments were approved by the Institutional Animal Care and Use Committee of Zhejiang cancer hospital (2022-08-005). For detailed animal experimental procedures and analysis methods, please refer to the Supplementary Data S1.

### Haematoxylin-eosin staining and immunohistochemistry

Briefly, all tissues were fixed in paraformaldehyde, dehydrated in ethanol, cleared with xylene, and paraffin-embedded. An H&E staining kit was used to stain the tissue slices. For immunohistochemical staining, the samples were incubated with antibodies. The sections were then incubated with biotin-labelled goat-rabbit IgG and horseradish peroxidase-conjugated streptavidin for 1 h. H&E staining and immunohistochemistry were then performed with an inverted microscope at 200× magnification. For detailed experimental and analytical methods, please refer to the Supplementary Data S1.

### TEM

Approximately 1 × 10^6^ AGS and MKN1 cells were seeded into a 6-well plate and cocultured with bacteria for 2 h (MOI = 10). Following cell digestion, the cells were fixed using 2.5% glutaraldehyde. Subsequently, the cells were further fixed with 1% osmic acid fixative solution, gradually dehydrated in an ascending ethanol concentration series, and embedded in epoxy resin. Sections (50–70 nm) were sliced, stained with 2% uranyl acetate and lead citrate, and imaged using a JEM-2100plus transmission electron microscope.

### EDU experiment

For the coculture of bacteria and cells, MKN1 and AGS cells were seeded in a 24-well plate at a density of 5 × 10^3^ cells per well. After overnight incubation, the cells were cocultured with SA for 2 h and then switched to complete RPMI 1640 medium for 3 days of further culture. For coculture with metabolites, MKN1 and AGS cells were seeded in a 24-well plate at a density of 5 × 10^3^ cells per well. After overnight incubation, the cells were cultured in low-serum RPMI 1640 medium supplemented with 1% BHI or 1% SAM for 3 days. Cell proliferation was assessed using the BeyoClick™ EdU-594 Cell Proliferation Assay Kit, and images were captured at 200X magnification using an inverted fluorescence microscope.

### Wound-healing assay

For coculture of bacteria and cells, MKN1 and AGS cells were seeded in a cell culture insert at a density of 1 × 10^5^ cells/chamber. After incubation overnight, the cell culture inserts were removed using sterile tweezers, after which the cells were rinsed with PBS three times and then cocultured with SA for 2 h. For metabolite coculture, MKN1 and AGS cells were seeded at a density of 1 × 10^5^ cells/chamber. After incubation overnight, the cell culture inserts were removed using sterile tweezers and then washed with PBS three times. The cells were cultured in low-serum RPMI 1640 medium supplemented with 1% BHI or 1% SAM. Images were captured at 0 h and 24 h using a CKX53 Olympus inverted fluorescence microscope.

### Transwell invasion (Matrigel) experiment

For coculture of bacteria and cells, MKN1 and AGS cells were seeded at 1 × 10^6^ cells per well in a 6-well plate and cocultured with SA for 2 h (MOI = 0, 10, 25, 50). Transwell chambers (Corning) were used, with 200 μL of cell suspension added to the upper chamber and 700 μL of medium containing 10% foetal bovine serum added to the lower chamber. It is then fixed, dyed and photographed. For metabolite coculture, the cells were digested with trypsin and centrifuged, and the cell concentrations were adjusted to 1 × 10^5^ cells/mL for MKN1 cells and 2 × 10^5^ cells/mL for AGS cells using serum-free base medium. The other steps were similar to those used for the coculture of bacteria and cells. For detailed experimental and analytical methods, please refer to the Supplementary Data S1.

### Flow cytometry

Whole blood was collected from healthy individuals, and PBMCs were isolated using Ficoll-Paque density gradient centrifugation. Then, SA was added to the upper compartment of the chamber to stimulate the PBMCs for 48 h. After that, the PBMCs were stained with APC-conjugated mouse anti-human CD8 and FITC-conjugated mouse anti-human CD3 in staining buffer and analyzed using a flow cytometer. For detailed experimental and analytical methods, please refer to the Supplementary Data S1.

### Bacterial arginine dihydrolase experiment

To assess the arginine metabolism capability of SA, 2.5 × 10^8^ CFU of SA with different concentrations of the ADI inhibitor L-CAV (0 mM, 0.5 mM, 1 mM, 2 mM) was added to 1 mL of arginine dihydrolase broth (Haibo Biotechnology Co., Ltd., Qingdao, China). The colour changes were captured at 0 h, 6 h, 9 h, 12 h, 15 h, 18 h, 21 h, and 24 h.

### LC‒MS/MS detection of ornithine and arginine in tumour and gastric tissues

Approximately 30 mg of mouse tumour tissue or MNU model stomach tissue was collected, homogenized and methanol was added to precipitate proteins, and samples were pre-treated for mass spectrometry analysis. Detailed steps for mass spectrometry analysis are described in the Supplementary Data S1.

### The ability of SA to metabolize arginine

SA was co-cultured with arginine to examine the metabolic level of arginine at different times (0, 3, 6, 12, or 24 h); meanwhile, the metabolic function of SA was further examined by adding the inhibitor L-CAV. Detailed methods for arginine assay are described in the Supplementary Data S1.

### Mass spectrometry imaging technology

Gastric cancer tissue is first frozen sectioned, and the sections were derivatised and matrix sprayed. The matrix-coated slides were then analyzed using an imaging mass spectrometry microscope, and ion extraction was performed for the derivatized products of the target metabolites. The target ion for derivatized arginine was M + H^+^. The imaging data were processed using IMAGEREVEAL MS software. The detailed experimental procedures and mass spectrometry parameters are described in the Supplementary Data S1.

### FISH

Gastric cancer sections were stained for FISH, and fluorescence microscopy was used to observe and take pictures, and the specific staining steps are detailed in the Supplementary Data S1.

## Supplementary information

Supplementary_Data S1

Supplementary Tables S1–S9

Supplementary Fig. S1

Supplementary Fig. S2

Supplementary Fig. S3

Supplementary Fig. S4

Supplementary Fig. S5

Supplementary Fig. S6

Supplementary Fig. S7

Supplementary Fig. S8

## Data Availability

The genomic information of *Streptococcus anginosus*, and the raw reads of 16S rRNA gene metabarcoding and the raw data of transcriptome were deposited into the NCBI SRA database (Accession Number: Bioproject PRJNA1061213). The raw data of metabolome deposited into the MetaboLights database (Accession Number: MTBLS9211). The code used in this study and all supporting data are available upon request.
