# Supplementary material for: Characterization of the landscape of the intratumoral microbiota reveals that Streptococcus anginosus increases the risk of gastric cancer initiation and progression
Source: Cell Discov. 2024 Nov 26;10:117. doi: 10.1038/s41421-024-00746-0 (PMC11589709; doi:10.1038/s41421-024-00746-0)
Supplement: Supplementary file 1 — Supplementary_Data S1 [file 41421_2024_746_MOESM1_ESM.pdf]

Supplementary Data S1

Materials and Methods

16S rRNA sequencing

Microbial DNA was extracted using an E.Z.N.A. Tissue DNA Kit (D3396-01; Omega, Norcross, Georgia, USA) following the manufacturer's instructions, as described previously. The DNA was quantified using a Qubit 2.0 fluorometer (Invitrogen, Carlsbad, CA, USA), and the molecular size was estimated via agarose gel electrophoresis. Primers targeting the hypervariable V3-V4 region of the 16S rRNA gene were used to amplify the extracted DNA samples. The forward primer sequence was 5'-CCTACGGGNGGCWGCAG-3', and the reverse primer sequence was 5'-GACTACHVGGGTATCTAATCC-3'. An AxyPrep PCR Clean-up Kit (AP-PCR-500G; Corning, NY, USA) was used to separate, extract and purify the PCR products, and the products were quantified using a Quant-iT PicoGreen dsDNA Reagent (P7581; Thermo Scientific, Waltham, MA, USA). After quality determination, libraries passing quality control were subjected to 2x two-terminal sequencing of 250 bp on a NovaSeq sequencer at LC-Bio Co., Ltd.

Ward’s hierarchical clustering analysis was utilized for sample categorization. Alpha diversity was assessed using the Shannon index with the vegan R package[1]. Beta diversity analysis between sample clusters was conducted by calculating Bray–Curtis distances with the vegan R package[1] and conducting principal coordinates analysis (PCoA) using the ape R package[2]. Survival analysis was performed using the Kaplan–Meier method with the survival R package[3, 4].

RNA-seq assays

mRNA sequencing (RNA-seq) was performed on paired tumour tissue and normal tissue samples from 108 AEG patients. Total RNA was isolated from the tumour tissue and normal tissue samples in RNA protective solution using TRIzol reagent (Invitrogen, Carlsbad, CA, USA) following the manufacturer's procedure. The RNA

concentration and purity of each sample were quantified by using a NanoDrop ND-1000 (NanoDrop, Wilmington, DE, USA). The RNA integrity was assessed with an Agilent 2100 with a RIN >7.0. For mRNA sequencing, a library was prepared from 1 μg of DNase I-treated total RNA using a TruSeq kit (Illumina), and 150-bp paired-end sequencing was performed on an Illumina HiSeq X Ten machine at LC-Bio Technology Co., Ltd. (Hangzhou, China), following the vendor's recommended protocol.

We aligned the reads of all the samples to the < research species > reference genome using the HISAT2 (https://daehwankimlab.github.io/hisat2/, version: hisat2-2.0.4) package, which initially removes a portion of the reads based on quality information accompanying each read and subsequently maps the reads to the reference genome[5, 6]. HISAT2 allows multiple alignments per read (up to 20 by default) and a maximum of two mismatches when mapping the reads to the reference. HISAT2 builds a database of potential splice junctions and confirms these findings by comparing the previously unmapped reads against the database of putative junctions. The mapped reads of each sample were assembled using StringTie (http://ccb.jhu.edu/software/stringtie/, version:stringtie- 1.3.4d) with default parameters. Then, all the transcriptomes from all the samples were merged to construct a comprehensive transcriptome using gffcompare software (http://ccb.jhu.edu/software/stringtie/gffcompare.shtml, version gffcompare-0.9.8)[6]. After the final transcriptome was generated, StringTie and Ballgown (http://www.bioconductor.org/packages/release/bioc/html/ballgown.html) were used to estimate the expression levels of all the transcripts and determine the expression abundance of the mRNAs by calculating the fragment per kilobase of transcript per million mapped reads (FPKM) value[7].

Differentially expressed genes (DEGs) were identified with DESeq2[8]. Genes with a FC ≥ 2 and an adjusted p value < 0.05 were considered to be significant DEGs. Gene set enrichment analysis (GSEA)[9, 10] was used to elucidate enriched functional pathways and modules, with an adjusted p value < 0.05 and $|NES| > 1$ as thresholds of

significance. CIBERSORT[11] was used to predict immune cell infiltration abundances. The Wilcoxon test was used to examine differences between sample groups.

Metabolomic assays

The samples were removed from the –80 °C freezer and thawed on ice, after which the metabolites were extracted with 80% methanol buffer. Briefly, 50 mg of sample was extracted with 0.5 ml of prechilled 80% methanol. The extraction mixture was then stored for 30 min at –20 °C. After centrifugation at 20,000 × g for 15 min, the supernatants were transferred to new tubes and vacuum dried. The samples were redissolved in 100 μL of 80% methanol and stored at –80 °C prior to LC–MS analysis. In addition, pooled QC samples were prepared by combining 10 μL of each extraction mixture. The extracted samples were then sorted for machine analysis with randomization. QC samples were inserted before, in the middle of, and after the samples to evaluate the experimental technical replicates. The samples were subjected to positive and negative ion mass spectrometry scans. All samples were analysed with an LC–MS system following the manufacturer’s instructions. First, all chromatographic separations were performed using an UltiMate 3000 UPLC System (Thermo Fisher Scientific, Bremen, Germany). An ACQUITY UPLC T3 column (100 mm×2.1 mm, 1.8 μm; Waters, Milford, USA) was used for reverse-phase separation. The column temperature was maintained at 40 °C. A TripleTOF 6600 high-resolution tandem mass spectrometer (SCIEX, Framingham, MA, USA) was used to detect metabolites eluted from the column. The Q-TOF was operated in both positive and negative ion modes. The curtain gas was set to 30 PSI, the long source gas 1 was set to 60 PSI, the long source gas 2 was set to 60 PSI, and the interface heater temperature was 500 °C. For positive ion mode, the floating ionspray voltage was set at 5000 V. For negative ion mode, the floating ionspray voltage was set at -4500 V. The mass spectrometry data were acquired in IDA mode. The TOF mass range was 60 to 1200 Da. The survey scans were acquired in 150 ms, and as many as 12 production scans were collected if the scans exceeded a threshold of 100 counts/second (counts/s)

and had a 1^{+}charge-state. Dynamic exclusion was set for 4 s. During acquisition, the mass accuracy was calibrated every 20 samples. Furthermore, to evaluate the stability of the LC–MS during the whole acquisition, a quality control sample (pool of all samples) was acquired after every 10 samples.

The raw mass spectrometry data were converted into readable data in mzXML format using Proteowizard's MSConvert software. XCMS software was used for peak extraction, and peak extraction quality control was conducted. Subsequently, the extracted substances were annotated using CAMERA for adduct and ion annotation, followed by primary identification using metaX software. Identification was performed separately by using the first-level mass spectrometry information and matching the second-level mass spectrometry information with an in-house standard compound database. Differentially abundant metabolites were identified by the Wilcoxon test. Metabolites with a $FC \geq 1.3$ and an adjusted p value<0.05 were considered to be significantly different.

Culturomics-based isolation of single bacteria from GC tissue

The stomach tissue blocks were removed from the –80 °C freezer, thawed on ice, and placed in a sterile mortar. Sterile PBS was added, and the mixture was ground until it became a uniform homogenate. The homogenate was transferred to a 10 mL sterile centrifuge tube. One millilitre of the homogenate was subjected to a 1:10 gradient dilution (dilution gradient from 1 to 10^{7}). Dilution gradients of 10^{4}, 10^{5}, 10^{6}, and 10^{7} were selected, and 500 µl was removed from each dilution to spread on plates (one replicate was set for each gradient). All the plates were incubated at 30 °C for 48 hours and then transferred to 37 °C and incubated for another 48 hours. After observing the bacterial growth, colonies with distinct morphologies were picked and inoculated onto fresh Columbia blood agar plates (M0028B, Shandong Tuopu Biol-Engineering Co., Ltd.). The plates were divided into six sections and inoculated once in each section, and a number was assigned to each section. The mixture was incubated at 37 °C for 48 hours. The growth of the colonies was observed, and the sections in which colonies had identical growth were excluded Colonies with different

morphologies were transferred to separate Columbia blood agar plates and incubated at 37 °C for 48 hours. After a portion of the bacterial colony was collected, DNA was extracted, and 27F and 1429R were used as primers for PCR amplification. The amplified products were subsequently sent to Sangon Biotech for sequencing. The sequencing results were compared with those from the NCBI database to isolate and preserve single bacterial species.

Bacterial culture and total metabolic products of SA (SAMs)

SA obtained through a GC tissue culture-based omics approach was cultured in brain heart fusion broth (BHI) (Huankai Microbial Technology Co., Ltd., Guangzhou, China) at 37 °C. After SA was cultivated to a concentration of 1×10^9 CFU/mL, the bacterial culture medium was centrifuged at 12000 rpm for 15 minutes, the supernatant was collected, and the mixture was passed through a 0.22 μm filter with a pore size of m to obtain the SAMs.

Cell lines and cell culture

Human GC cell lines, including AGS and MKN1 cells, and the mouse MFC cell line were obtained from Cobioer Biosciences Co., Ltd. (Nanjing, China). AGS, MKN1 and MFC cells were cultured in RPMI 1640 medium (Kino Biological and Pharmaceutical Technology Co., Ltd., Hangzhou, China) supplemented with 10% foetal bovine serum (FBS; Gibco, Grand Island, USA) and 1% penicillin/streptomycin (Kino Co., Ltd., Hangzhou, China) at 37 °C in a 5% CO_2 incubator. These two cell lines were identified by short tandem repeats, and bacterial and fungal contamination tests were negative.

MNU-induced spontaneous GC model

The mice were divided into three groups, each comprising 12 male C57BL/6 mice aged 4-6 weeks: the control group, the MNU group, and the MNU+SA group. Initially, GC induction was performed on 24 of the mice using N-methyl-N-nitrosourea (MNU). All animal experiments were approved by the Institutional Animal Care and Use Committee of Zhejiang cancer hospital (2022-08-110). The

induction procedure involved the addition of 120 ppm MNU to the drinking water for one week, followed by one week of regular water consumption, constituting one cycle. This process was repeated for a total of five cycles, with a duration of 5 weeks for MNU induction and a total study duration of 10 weeks. After the induction period, a single bacterial intervention was introduced. The control and MNU groups were administered PBS, while the MNU+SA group was administered SA at a dose of 1×10^9 CFU/day via oral gavage three times per week at 0.2 mL/dose. This treatment was administered for 12 consecutive weeks, followed by gavage once every 4 weeks. The entire experiment spanned 44 weeks, during which 2 mice from the control group and MNU+SA group died and 1 mouse from the MNU group died. Weekly records of body weight were maintained throughout the experiment, and upon completion, the blood, stomach, intestines, and other organs were collected for further analysis.

Transplanted tumour model with subcutaneously transplanted MFC cells

The experiment comprised 5 groups, each with 6 male 615 mice aged 6-8 weeks, for a total of 30 mice. The groups included the control group, low-dose SA group, high-dose SA group, low-dose SA group treated with ceftriaxone (CFR), and high-dose SA group treated with CFR. All animal experiments were approved by the Institutional Animal Care and Use Committee of Zhejiang cancer hospital (2022-08-005). Subcutaneous inoculation of MFC GC cells (2.5×10^5/50 μL) resulted in tumour formation within approximately one week. After tumour formation, the mice were randomly grouped based on tumour size. The control group received an intratumoral injection of 50 μL of PBS, the low-dose SA group received an intratumoral injection of 50 μL of bacterial suspension (5×10^6 CFU, prepared at a concentration of 1×10^8 CFU/mL), the high-dose SA group received an intratumoral injection of 50 μL of bacterial suspension (5×10^7 CFU, prepared at a concentration of 1×10^9 CFU/mL), the low-dose SA+CFR group received an intratumoral injection of 50 μL of bacterial suspension (5×10^6 CFU, prepared at a concentration of 1×10^8 CFU/mL) combined with oral gavage of 200 mg/kg CFR, and the high-dose SA+CFR group received an intratumoral injection of 50 μL of bacterial suspension (5×10^7 CFU, prepared at a

concentration of 1×10^9 CFU/mL) combined with oral gavage of 200 mg/kg CFR once daily. Tumour size and body weight were monitored every other day, and the experiment lasted for 15 days or until the tumour size exceeded 1500 mm^3. Upon completion of the experiment, the blood and tumour tissues were collected for further analysis.

Haematoxylin-eosin staining and immunohistochemistry

Haematoxylin-eosin staining and immunohistochemistry were performed as described previously[12]. Briefly, all tissues were fixed in paraformaldehyde, dehydrated in ethanol, cleared with xylene, and paraffin-embedded. A haematoxylin-eosin (H&E) staining kit (Art. ZLI-9609 ZSGB-BIO Corp., Shanghai, China) was used to stain the tissue slices. For immunohistochemical staining, the samples were incubated with antibodies against CD8 (Cat. No.: ab17147), Ki-67 (Cat. No.: ab15580), N-cadherin (Cat. No.: ab76011) and vimentin (Cat. No.: ab20346) from Abcam (Cambridge, UK). The sections were then incubated with biotin-labelled goat-rabbit IgG and horseradish peroxidase-conjugated streptavidin for 1 hour. Haematoxylin-eosin staining and immunohistochemistry were then performed with an inverted microscope at 200× magnification.

Transmission electron microscopy (TEM)

Approximately 1×10^6 AGS and MKN1 cells were seeded into a 6-well plate and cocultured with bacteria for 2 hours (MOI=10). Following cell digestion, the cells were fixed using 2.5% glutaraldehyde. Subsequently, the cells were further fixed with 1% osmic acid fixative solution, gradually dehydrated in an ascending ethanol concentration series, and embedded in epoxy resin. Sections (50-70 nm) were sliced, stained with 2% uranyl acetate and lead citrate, and imaged using a JEM-2100plus transmission electron microscope.

5-Ethynyl-2'-deoxyuridine (EDU) experiment

For the coculture of bacteria and cells, MKN1 and AGS cells were seeded in a 24-well plate at a density of 5×10^3 cells per well. After overnight incubation, the cells

were cocultured with SA (MOI=0, 10, 25, 50) for 2 hours and then switched to complete RPMI 1640 medium for 3 days of further culture. For coculture with metabolites, MKN1 and AGS cells were seeded in a 24-well plate at a density of 5×10^3 cells per well. After overnight incubation, the cells were cultured in low-serum RPMI 1640 medium supplemented with 1% BHI or 1% SAM for 3 days. Cell proliferation was assessed using the BeyoClick™ EdU-594 Cell Proliferation Assay Kit (Biyuntian Biotechnology Co., Ltd., Shanghai, China), and images were captured at 200X magnification using an inverted fluorescence microscope.

Wound-healing assay

For coculture of bacteria and cells, MKN1 and AGS cells were seeded in a cell culture insert (Ibidi, Munich, Germany) at a density of 1×10^5 cells/chamber. After incubation overnight, the cell culture inserts were removed using sterile tweezers, after which the cells were rinsed with PBS three times and then cocultured with SA for 2 hours (MOI= 0, 10, 25, 50). For metabolite coculture, MKN1 and AGS cells were seeded at a density of 1×10^5 cells/chamber. After incubation overnight, the cell culture inserts were removed using sterile tweezers and then washed with PBS three times. The cells were cultured in low-serum RPMI 1640 medium supplemented with 1% BHI or 1% SAM. Images were captured at 0 h and 24 h using a CKX53 Olympus inverted fluorescence microscope.

Transwell invasion (Matrigel) experiment

For coculture of bacteria and cells, MKN1 and AGS cells were seeded at 1×10^6 cells per well in a 6-well plate and cocultured with SA for 2 hours (MOI=0, 10, 25, 50). The medium containing bacteria was removed, and the cells were washed three times with PBS. Trypsin was used to digest the cells, followed by centrifugation. The cell concentrations were adjusted to 1×10^5 MKN1 cells/mL and 2×10^5 AGS cells/mL using FBS-free RPMI 1640 medium. Transwell chambers (Corning) were used, with 200 μL of cell suspension added to the upper chamber and 700 μL of medium containing 10% foetal bovine serum added to the lower chamber. After 24 hours, the

chambers were removed from the incubator. The cells in the upper chamber were removed with a cotton swab, and those in the lower chamber were fixed with 4% paraformaldehyde and stained with crystal violet. Images were captured using a CKX53 Olympus inverted fluorescence microscope (200X). For metabolite coculture, the cells were digested with trypsin and centrifuged, and the cell concentrations were adjusted to 1×10^5 cells/mL for MKN1 cells and 2×10^5 cells/mL for AGS cells using serum-free base medium. Transwell chambers (Corning) were used, with 200 μL of cell suspension added to the upper chamber and 700 μL of medium containing 10% foetal bovine serum added to the lower chamber. After 24 hours of treatment (control, 1% BHI, 1% SAM), the chambers were removed from the incubator. The other steps were similar to those used for the coculture of bacteria and cells.

Flow cytometry

Whole blood was collected from healthy individuals, and PBMCs were isolated using Ficoll-Paque density gradient centrifugation (Sigma Aldrich, GE17-1440-03). Then, 1×10^5 PBMCs were seeded in a 24-well plate, and a Transwell chamber (0.4 µm pore) was placed in the 24-well plate. One hundred microlitres of medium was added to the upper chamber of the Transwell plate, and 700 μL of medium containing 10% FBS was added to the lower chamber. Then, SA was added to the upper compartment of the chamber to stimulate the PBMCs for 48 hours. After that, the PBMCs were stained with APC-conjugated mouse anti-human CD8 (BD Bioscience, 566852) and FITC-conjugated mouse anti-human CD3 (BD Bioscience, 555916) in staining buffer (BD Bioscience, 554656) and analysed using a flow cytometer (Thermo Fisher Scientific, MA, USA).

Bacterial arginine dihydrolase experiment

To assess the arginine metabolism capability of SA, 2.5×10^8 CFU of SA with different concentrations of the ADI inhibitor L-CAV (0 mM, 0.5 mM, 1 mM, 2 mM) was added to 1 mL of arginine dihydrolase broth (Haibo Biotechnology Co., Ltd.,

Qingdao, China). The colour changes were captured at 0 h, 6 h, 9 h, 12 h, 15 h, 18 h, 21 h, and 24 h.

LC–MS/MS detection of ornithine and arginine in tumour and gastric tissues

Chromatography and Mass Spectrometry: Data acquisition was performed using a Vanquish UHPLC instrument coupled with an Orbitrap Exploris 120 (Thermo Fisher Scientific, USA). The raw data were acquired using Xcalibur software. Liquid chromatographic separation was carried out on a Waters UPLC HSS T3 column (1.8 µm; 150 mm length × 2.1 mm internal diameter), and the column temperature was maintained at 40 °C. Mobile phase A consisted of water with 0.1% formic acid (LC–MS/MS grade; Thermo Fisher), and mobile phase B consisted of acetonitrile (LC–MS/MS grade; Thermo Fisher) at a flow rate of 0.3 mL/min. The gradient program was set as follows: 0–1 min, 2% B; 1–3 min, 2–60% B; 3–5 min, 60% B; 5–7 min, 60–100% B; 7–8 min, 100% B; 8–8.1 min, 100–2% B; and 10 min, 2% B. The injection volume was 10 µL. During data acquisition, all the samples were analysed randomly. Quality control (QC) samples were prepared by pooling equal aliquots of all samples and were injected after every 10 sample injections.

Data acquisition was performed in full scan mode and dd-MS2 scan mode. The ion source parameters were set as follows: spray voltage at 3500 V for positive mode, sheath gas at 50 arb, aux gas at 10 arb, and ion transfer tube temperature at 300 °C. The resolution for the full MS scan mode was set to 60,000, and the AGC target for the positive mode was set to 1e6. The maximum injection time was set to 100 milliseconds. The mass range was set to 60-900 Da. For dd-MS2 scan mode, the MS resolution was set to 15,000, and the AGC target was set to standard. The maximum injection time was set to Auto. The collision energy was set to SNCE 20-40-60%. The [M+H]+ of ornithine was 133.0970, with a retention time of 0.705 min; the [M+H]+ of arginine was 175.1189, with a retention time of 0.818 min. All retention times were compared using standard substances.

Approximately 30 mg of mouse tumour tissue or stomach tissue from the MNU model was collected, steel beads and 5 volumes of pure water were added, and the tissues were ground using a tissue homogenizer (Model: KZ-5F-3D, 3D Cryogenic Grinder, Servicebio) until a homogeneous mixture was obtained. One hundred microlitres of the homogenate was added, 300 µL of methanol was added to precipitate the proteins, and the mixture was incubated at –20 °C for 1 hour. Then, the samples were centrifuged at 12000 rpm for 15 minutes. Three hundred microlitres of the supernatant was collected and lyophilized using a Labconco Corporation Labconco freeze dryer. Finally, the cells were resuspended in 100 µL of methanol:water (1:1) and centrifuged, after which the supernatant was collected for injection.

The ability of SA to metabolize arginine

SA was inoculated into BHI culture medium and incubated at 37 °C with shaking at 200 rpm for 12 hours. The OD600 of the bacterial strains was determined using a spectrophotometer. After centrifugation at 4 °C and 12000 rpm for 10 min, the supernatant was removed, and the bacterial pellet was resuspended in PBS. The bacterial concentration was quantified at 5×10^8 CFU/mL, after which L-arginine was added (to a final concentration of 25 mM). The bacterial strain was cocultured with L-arginine for 0, 3, 6, 12, or 24 hours. To elucidate the potential of L-CAV to competitively inhibit arginine metabolism in SA, SA was cocultured with both L-arginine (5 mM) and various concentrations of L-CAV (0 mM, 6 mM, 12 mM, 24 mM) for 3 h. Sample extraction was subsequently performed for mass spectrometry analysis.

For protein precipitation, 200 µL of SA cultured with L-arginine was collected and 600 µL of methanol was added, or 20 µL of the SA cocultured with both L-arginine and L-CAV was collected and 4 mL of methanol was added. The mixture was incubated at -80 °C for 1 hour and then centrifuged at 12000 rpm for 15 minutes. The supernatant was collected for injection, with a sample volume of 10 µL. The mass spectrometry method and instrument conditions were previously described. The liquid

chromatography parameters were as follows: 0–1 min, 2% B; 1–4.5 min, 2–60% B; 4.5–7 min, 60% B; 7–8 min, 60–100% B; 8–11.4 min, 100% B; 11.4–11.5 min, 100–2% B; and 14 min, 2% B.

Mass spectrometry imaging technology

The GC tissue sections were retrieved from the -80 °C freezer and placed in a desiccator for drying at room temperature for 15 minutes. A pretreatment method based on 3-nitrophenylhydrazine (3-NPH) derivatization[13] was employed, involving the spraying of 3-NPH (175 mM, Sigma), 1-(3-dimethylaminopropyl)-3 -ethylcarbodiimide hydrochloride (EDC, 105 mM, Sigma), and pyridine (dissolved in 2.5% methanol) onto the tissue section to increase sensitivity to amino acid-like small molecules. The matrix coating was 9-aminoacridine (9-AA, 10 mg/mL; Sigma–Aldrich). Both the derivatization and matrix application processes were carried out using an automated matrix sublimation device (iMLayer: SHIMADZU, Shimadzu Corporation). The matrix-coated slides were then analysed using an imaging mass spectrometry microscope (iMScope QT: SHIMADZU, Shimadzu Corporation), and ion extraction was performed for the derivatized products of the target metabolites (arginine and ornithine). The target ion for derivatized arginine was $M+H^+$ (m/z 310.1622), and for derivatized ornithine, it was $M+K^+$ (m/z 306.0969). The imaging data were processed using IMAGEREVEAL MS software (SHIMADZU, Shimadzu Corporation). Concurrently, the tissue sections were optically photographed at various magnifications, and a clear microscopy image of the tumour tissue was obtained under a 5x objective lens. The detailed settings of the imaging mass spectrometry microscope (iMScope QT: SHIMADZU, Shimadzu Corporation) were as follows: laser, 355 nm YAG laser; analysis mode, positive ion; pixel spacing, 10 μm × 10 μm; laser spot diameter, 10 μm; laser energy, 70 (range 0-100); number of laser shots, 200 shots; scanning frequency, 5000 Hz; scanning range, 70-500; and detector voltage, 2.40 kV.

FISH

Paraffin sections were deparaffinized by immersing them twice in xylene for 10 minutes each. The sections were sequentially rehydrated in 100%, 85%, and 75% ethanol for 3 minutes each, followed by incubation in PBS for 3 minutes. The specimens were then incubated in 0.2 mol/L hydrochloric acid for 12 minutes at 37 °C. The specimens were washed in PBS for 3 minutes. Then, the specimens were incubated in quercetin for 12 minutes at 37 °C, followed by incubation in lysozyme solution for another 12 minutes at the same temperature. The specimens were washed twice in PBS for 5 minutes each. The sections were air-dried. For prehybridization, the hybridization solution was removed from the refrigerator 30 minutes in advance to come to room temperature, the solution was added to the sections, and the sections were incubated in a hybridization instrument at 55 °C for 1 hour. The probe was diluted with bacterial hybridization solution at a ratio of 1:50, denatured at 85 °C for 3 minutes, and then equilibrated at 37 °C for 5 minutes. The prehybridized sections were removed, the excess liquid was discarded, and the probe was applied to the sections, ensuring complete coverage of the tissue. The mixture was incubated overnight in a hybridization instrument at 37 °C. The sections were washed three times in 2× SGS (0.1% NP-40) for 5 minutes each. The sections were dehydrated in an ethanol gradient and mounted with DAPI. The cells were observed under a fluorescence microscope. The probe was synthesized by Exonbio (Gungzhou, China). For *Streptococcus* spp., the FISH probe was labelled with FITC:5'-CACTCTCCCCTTCTGCAC-3' [14] . The probe was purchased from Exonbio (Gungzhou, China).

References

1 Liu C, Cui Y, Li X, Yao M. microeco: an R package for data mining in microbial community ecology. FEMS microbiology ecology 2021;**97**.

2 Paradis E, Schliep K. ape 5.0: an environment for modern phylogenetics and evolutionary analyses in R. Bioinformatics (Oxford, England) 2019;**35**:526-8.

3 Sonabend R, Király FJ, Bender A, Bischl B, Lang M. mlr3proba: an R package for machine learning in survival analysis. Bioinformatics (Oxford, England) 2021;**37**:2789-91.

4 In J, Lee DK. Survival analysis: part II - applied clinical data analysis. Korean journal of anesthesiology 2019;**72**:441-57.

5 Kim D, Paggi JM, Park C, Bennett C, Salzberg SL. Graph-based genome alignment and genotyping with HISAT2 and HISAT-genotype. Nature biotechnology 2019;**37**:907-15.

6 Pertea M, Kim D, Pertea GM, Leek JT, Salzberg SL. Transcript-level expression analysis of RNA-seq experiments with HISAT, StringTie and Ballgown. Nature protocols 2016;**11**:1650-67.

7 Pertea M, Pertea GM, Antonescu CM, Chang TC, Mendell JT, Salzberg SL. StringTie enables improved reconstruction of a transcriptome from RNA-seq reads. Nature biotechnology 2015;**33**:290-5.

8 Love MI, Huber W, Anders S. Moderated estimation of fold change and dispersion for RNA-seq data with DESeq2. Genome biology 2014;**15**:550.

9 Subramanian A, Tamayo P, Mootha VK, Mukherjee S, Ebert BL, Gillette MA, *et al.* Gene set enrichment analysis: a knowledge-based approach for interpreting genome-wide expression profiles. Proceedings of the National Academy of Sciences of the United States of America 2005;**102**:15545-50.

10 Mootha VK, Lindgren CM, Eriksson KF, Subramanian A, Sihag S, Lehar J, *et al.* PGC-1alpha-responsive genes involved in oxidative phosphorylation are coordinately downregulated in human diabetes. Nature genetics 2003;**34**:267-73.

11 Newman AM, Liu CL, Green MR, Gentles AJ, Feng W, Xu Y, *et al.* Robust enumeration of cell subsets from tissue expression profiles. Nature methods 2015;**12**:453-7.

12 Li S, Yuan L, Xu ZY, Xu JL, Chen GP, Guan X, *et al.* Integrative proteomic characterization of adenocarcinoma of esophagogastric junction. Nature communications 2023;**14**:778.

13 Meng X, Pang H, Sun F, Jin X, Wang B, Yao K, *et al.* Simultaneous 3-Nitrophenylhydrazine Derivatization Strategy of Carbonyl, Carboxyl and Phosphoryl Submetabolome for LC-MS/MS-Based Targeted Metabolomics with Improved Sensitivity and Coverage. Analytical chemistry 2021;**93**:10075-83.

14 Trebesius, K., Leitritz, L., Adler, K. *et al.* Culture independent and rapid identification of bacterial pathogens in necrotising fasciitis and streptococcal toxic shock syndrome by fluorescence in situ hybridisation. Med Microbiol Immunol 2000;**188**, 169–175.
