# Supplementary material for: Characterization of the landscape of the intratumoral microbiota reveals that Streptococcus anginosus increases the risk of gastric cancer initiation and progression
Source: Cell Discov. 2024 Nov 26;10:117. doi: 10.1038/s41421-024-00746-0 (PMC11589709; doi:10.1038/s41421-024-00746-0)
Supplement: Supplementary file 3 — Supplementary Fig. S1 [file 41421_2024_746_MOESM3_ESM.pdf]

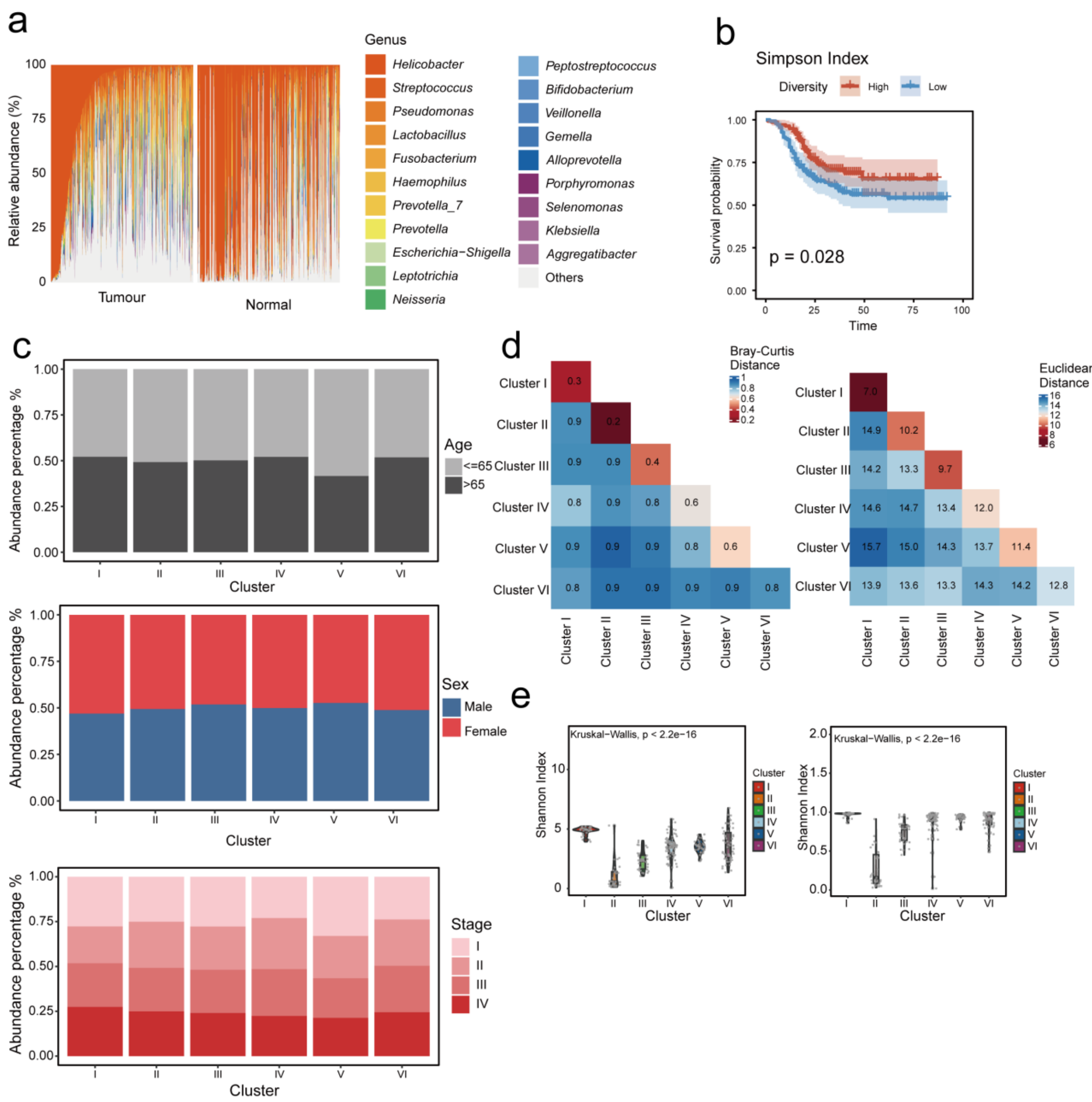

**Fig. S1 Differentiation of the intratumoral microbiota in GC. (a)** Microbiome composition of tumour and paired normal samples. **(b)**There is a significant correlation between higher alpha diversity in the tumor microbiome and improved patient prognosis. **(c)** Clinical information, including age, sex and stage, for the different clusters. **(d)** Euclidean distance within clusters calculated by CLR-transformed data and Beta-diversity within clusters represented by Bray-Curtis distance. **(e)** Alpha diversity of the different clusters. The Kruskal–Wallis and Wilcoxon tests were used to evaluate differences.
