# Supplementary material for: Characterization of the landscape of the intratumoral microbiota reveals that Streptococcus anginosus increases the risk of gastric cancer initiation and progression
Source: Cell Discov. 2024 Nov 26;10:117. doi: 10.1038/s41421-024-00746-0 (PMC11589709; doi:10.1038/s41421-024-00746-0)
Supplement: Supplementary file 4 — Supplementary Fig. S2 [file 41421_2024_746_MOESM4_ESM.pdf]

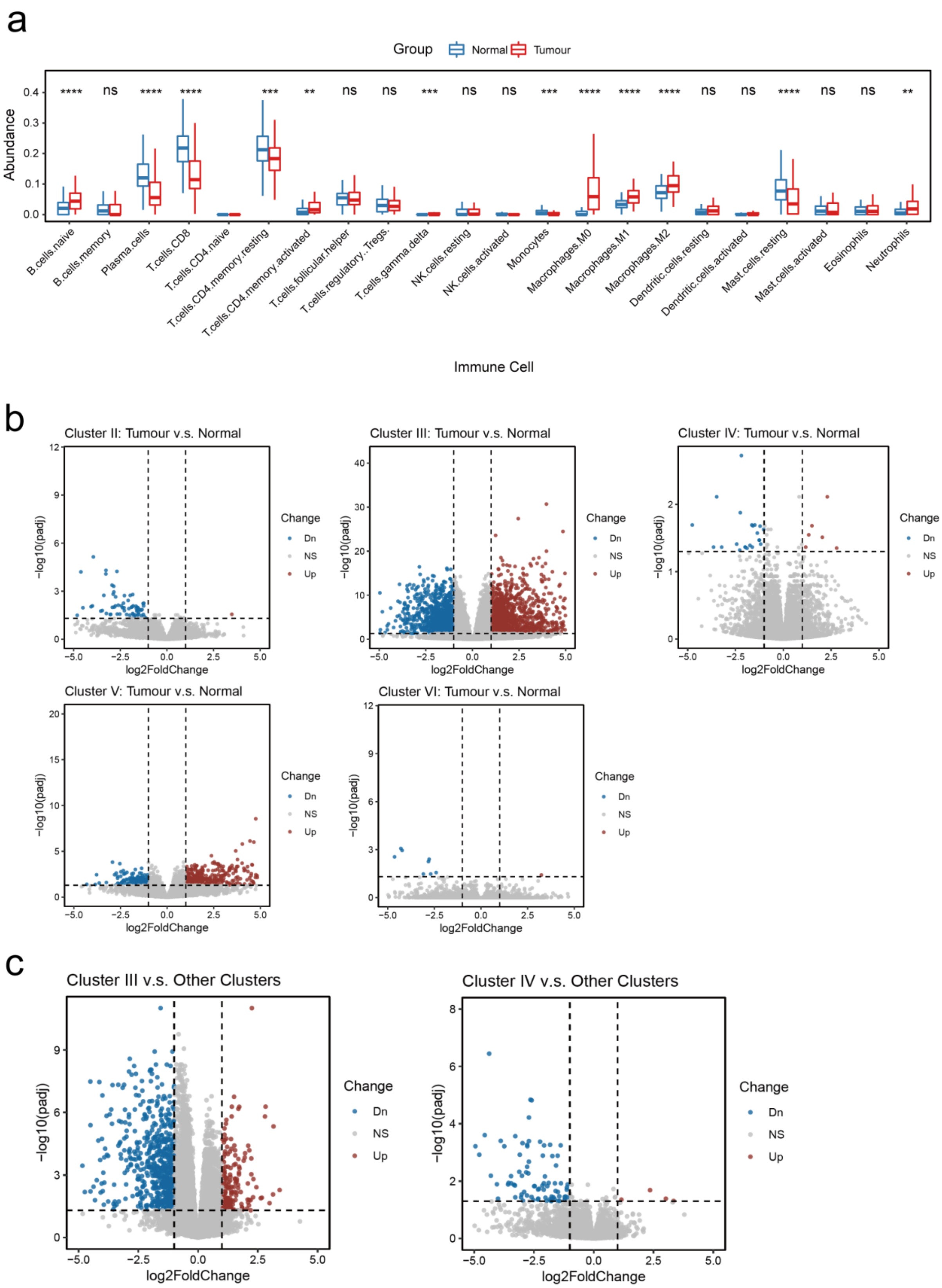
a
Group
Normal
Tumour
Abundance
0.4
0.3
0.2
0.1
0.0
**** ns **** **** *** ** ns ns *** ns ns *** **** **** **** ns ns **** ns ns **
B.cells.naive
B.cells.memory
Plasma.cells
T.cells.CD8
T.cells.CD4.naive
T.cells.CD4.memory.resting
T.cells.CD4.memory.activated
T.cells.follicular.helper
T.cells.regulatory..Tregs.
T.cells.gamma.delta
NK.cells.resting
NK.cells.activated
Monocytes
Macrophages.M0
Macrophages.M1
Macrophages.M2
Dendritic.cells.resting
Dendritic.cells.activated
Mast.cells.resting
Mast.cells.activated
Eosinophils
Neutrophils
Immune Cell
b
Cluster II: Tumour v.s. Normal
Cluster III: Tumour v.s. Normal
Cluster IV: Tumour v.s. Normal
Cluster V: Tumour v.s. Normal
Cluster VI: Tumour v.s. Normal
−log10(padj)
log2FoldChange
Change
Dn
NS
Up
c
Cluster III v.s. Other Clusters
Cluster IV v.s. Other Clusters
−log10(padj)
log2FoldChange
Change
Dn
NS
Up

Fig. S2 Bacteria regulate the expression of host genes and the infiltration of immune cells. **(a)** Predicted immune cell infiltration levels in tumour versus normal samples calculated by CIBERSORT. Significance threshold: adjusted P value < 0.05. **(b)** Differential expression analysis demonstrating genes differentially expressed between tumour and normal tissues within clusters III-VI. The significance thresholds were as follows: adjusted P value < 0.05 and fold change > 2. **(c)** Differential expression analysis demonstrating DEGs between cluster III/IV (tumour) and other clusters (tumour). The significance thresholds were as follows: adjusted P value < 0.05 and fold change > 2.
