# Supplementary material for: Characterization of the landscape of the intratumoral microbiota reveals that Streptococcus anginosus increases the risk of gastric cancer initiation and progression
Source: Cell Discov. 2024 Nov 26;10:117. doi: 10.1038/s41421-024-00746-0 (PMC11589709; doi:10.1038/s41421-024-00746-0)
Supplement: Supplementary file 5 — Supplementary Fig. S3 [file 41421_2024_746_MOESM5_ESM.pdf]

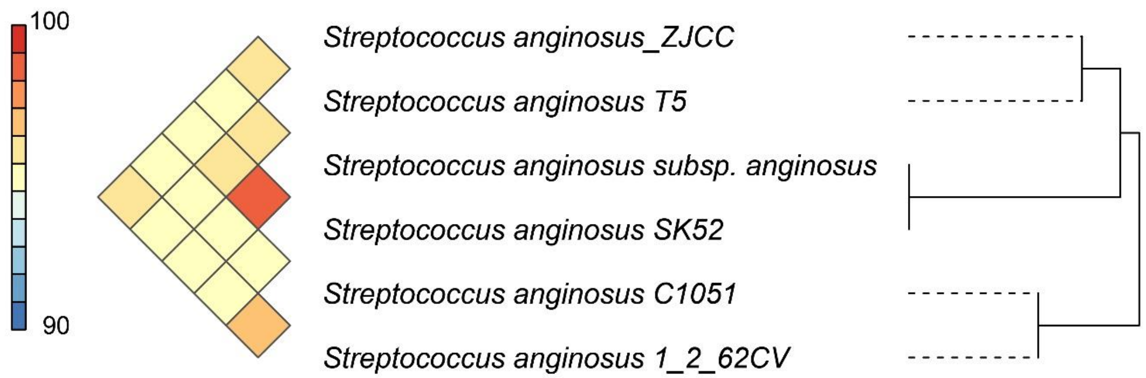

Fig. S3 Comparison of clinical isolate Streptococcus anginosus_ZJCC (This is the strain of bacteria we isolated from patient tissue) with the reference strain using Average Nucleotide Identity (ANI).
