# Supplementary material for: Characterization of the landscape of the intratumoral microbiota reveals that Streptococcus anginosus increases the risk of gastric cancer initiation and progression
Source: Cell Discov. 2024 Nov 26;10:117. doi: 10.1038/s41421-024-00746-0 (PMC11589709; doi:10.1038/s41421-024-00746-0)
Supplement: Supplementary file 7 — Supplementary Fig. S5 [file 41421_2024_746_MOESM7_ESM.pdf]

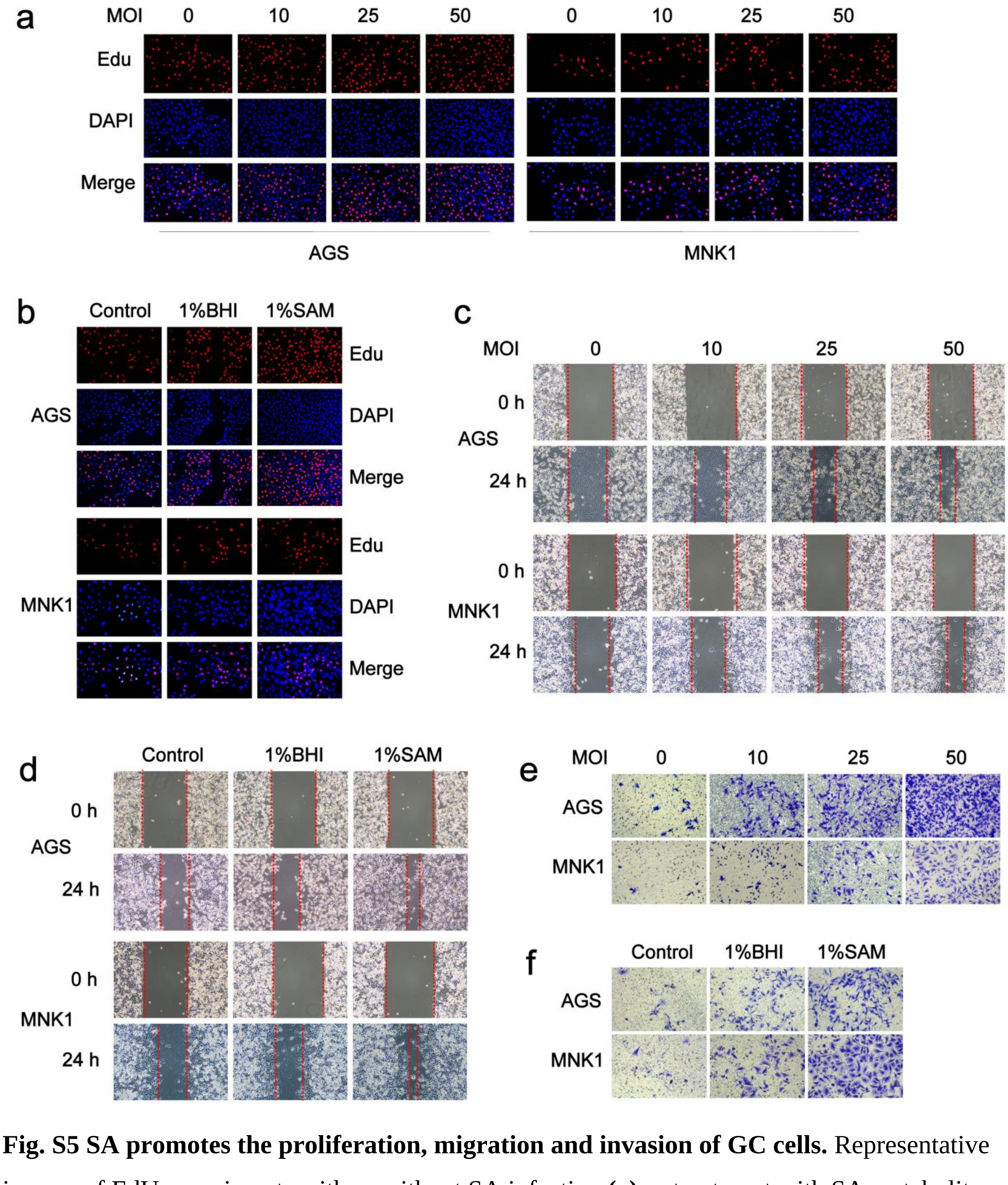

Fig. S5 SA promotes the proliferation, migration and invasion of GC cells. Representative images of EdU experiments with or without SA infection **(a)** or treatment with SA metabolites **(b)**. Representative images of wound healing experiments with or without SA infection **(c)** or

treatment with SA metabolites **(d).** Representative images of Transwell invasion experiments with or without SA infection **(e)** or treatment with SA metabolites **(f).**
