# Supplementary material for: Characterization of the landscape of the intratumoral microbiota reveals that Streptococcus anginosus increases the risk of gastric cancer initiation and progression
Source: Cell Discov. 2024 Nov 26;10:117. doi: 10.1038/s41421-024-00746-0 (PMC11589709; doi:10.1038/s41421-024-00746-0)
Supplement: Supplementary file 8 — Supplementary Fig. S6 [file 41421_2024_746_MOESM8_ESM.pdf]

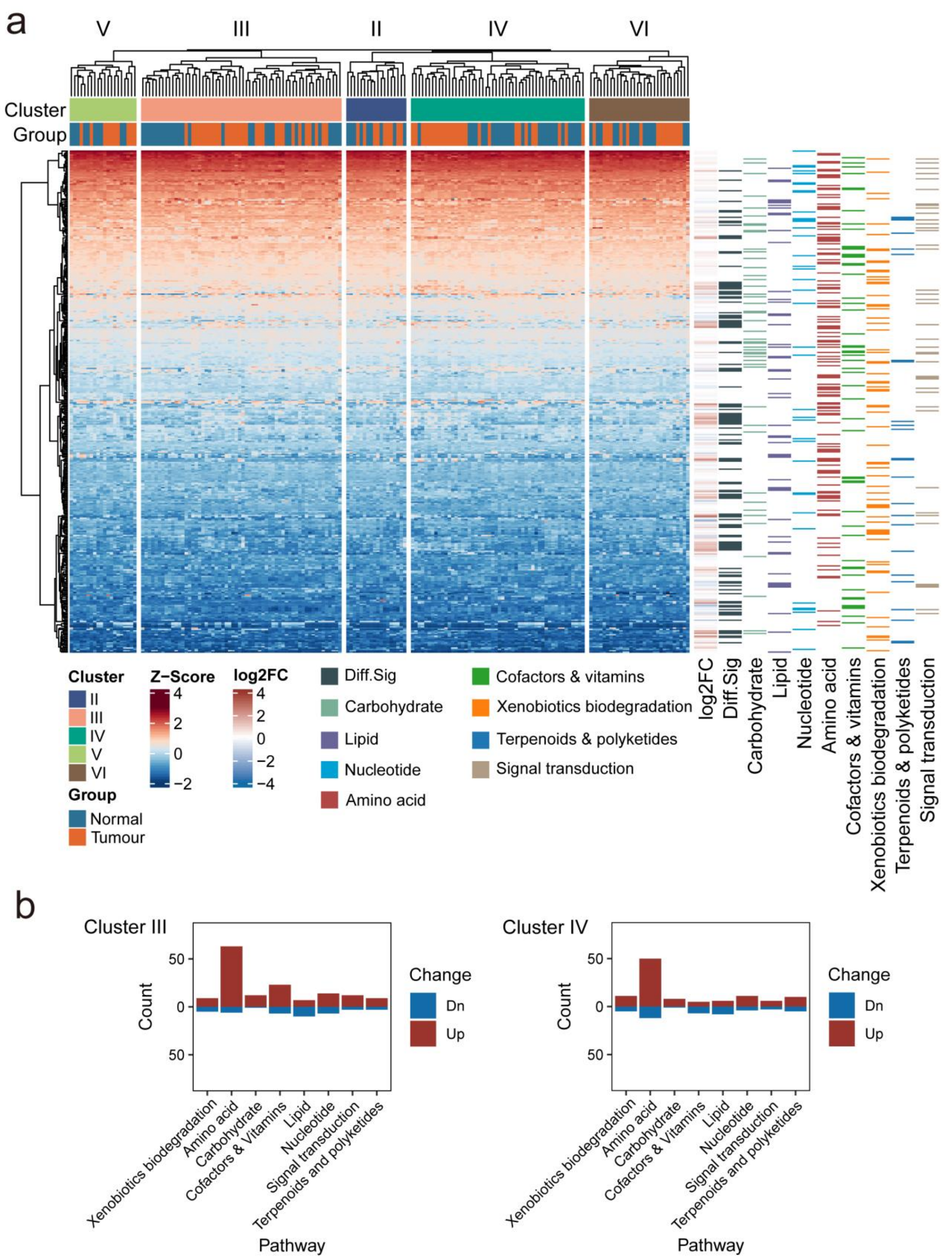

a
V
III
II
IV
VI
Cluster
Group
Cluster
II
III
IV
V
VI
Group
Normal
Tumour
Z−Score
4
2
0
−2
log2FC
4
2
0
−2
−4
Diff.Sig
Carbohydrate
Lipid
Nucleotide
Amino acid
Cofactors & vitamins
Xenobiotics biodegradation
Terpenoids & polyketides
Signal transduction
log2FC
Diff.Sig
Carbohydrate
Lipid
Nucleotide
Amino acid
Cofactors & vitamins
Xenobiotics biodegradation
Terpenoids & polyketides
Signal transduction
b
Cluster III
Count
50
0
50
Change
Dn
Up
Xenobiotics biodegradation
Amino acid
Carbohydrate
Cofactors & Vitamins
Lipid
Nucleotide
Signal transduction
Terpenoids and polyketides
Pathway
Cluster IV
Count
50
0
50
Change
Dn
Up
Xenobiotics biodegradation
Amino acid
Carbohydrate
Cofactors & Vitamins
Lipid
Nucleotide
Signal transduction
Terpenoids and polyketides
Pathway

Fig. S6 Compared to normal tissues, gastric cancer tissues exhibit significant metabolic changes. (a) Metabolite landscape representing differentially abundant metabolites within different clusters annotated by metabolite-related pathway. The numbers of differentially abundant metabolites in each pathway are displayed (top right). Significance thresholds: adjusted P value < 0.05 and fold change > 1.3. **(b)** Differentially abundant metabolites classified by their related pathways in cluster III and cluster IV. Significance thresholds: adjusted P value < 0.05 and fold change > 1.3.
