# Supplementary figures and images for: Characterization of the landscape of the intratumoral microbiota reveals that Streptococcus anginosus increases the risk of gastric cancer initiation and progression
Source: Cell Discov. 2024 Nov 26;10:117. doi: 10.1038/s41421-024-00746-0 (PMC11589709; doi:10.1038/s41421-024-00746-0)

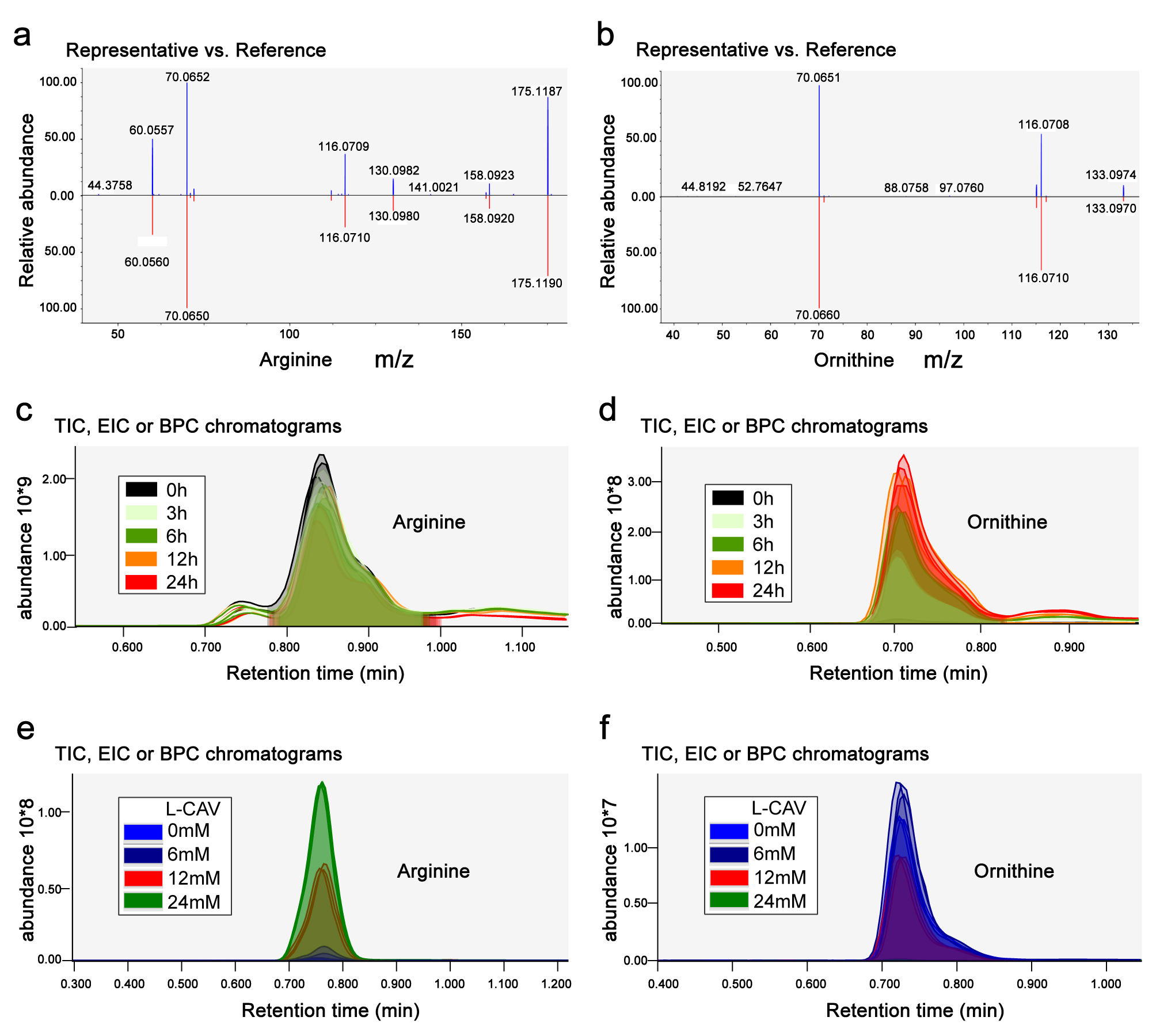

Supplement: Supplementary file 9 — Supplementary Fig. S7 [file 41421_2024_746_MOESM9_ESM.tif]
