# Supplementary material for: Characterization of the landscape of the intratumoral microbiota reveals that Streptococcus anginosus increases the risk of gastric cancer initiation and progression
Source: Cell Discov. 2024 Nov 26;10:117. doi: 10.1038/s41421-024-00746-0 (PMC11589709; doi:10.1038/s41421-024-00746-0)
Supplement: Supplementary file 10 — Supplementary Fig. S8 [file 41421_2024_746_MOESM10_ESM.pdf]

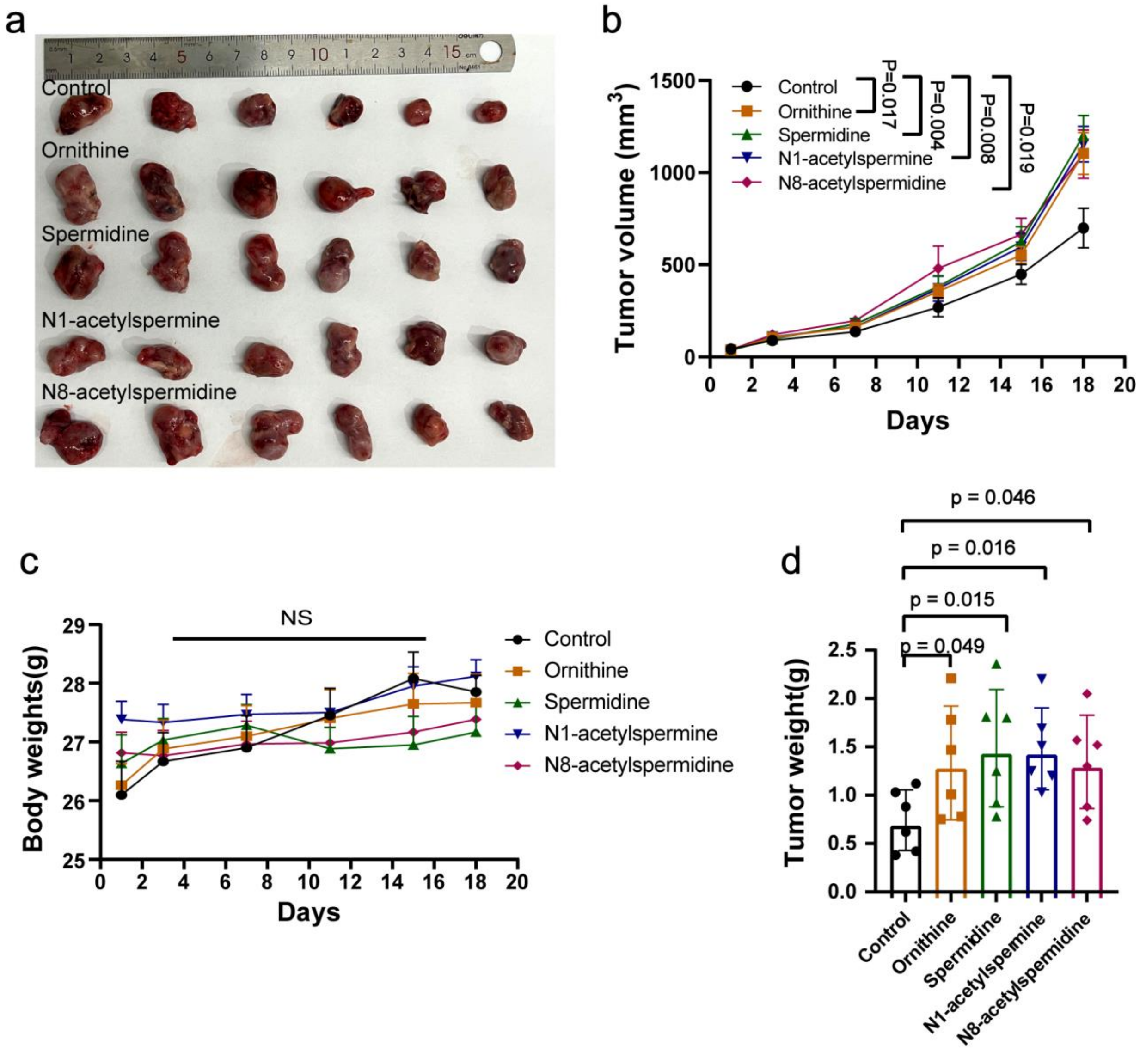

Fig. S8 The activation of arginine and its downstream metabolic pathways can promote the growth of gastric cancer. (a) Image showing the tumour sizes in each group in the mouse xenograft experiment. **(b)** Tumour volumes in each group in the mouse xenograft experiment. **(c)** Body weight in each group in the mouse xenograft experiment. **(d)** Tumour weight in each group in the mouse xenograft experiment.
